# Classification of outcomes in antimalarial therapeutic efficacy studies with Aster

**DOI:** 10.1101/2025.04.28.651146

**Published:** 2025-05-01

**Authors:** Inna Gerlovina, Sophie Berube, Jessica Briggs, Kathryn Murie, Maxwell Murphy, Amy Wesolowski, Bryan Greenhouse

**Affiliations:** 1 EPPIcenter research program, Division of HIV, ID and Global Medicine, Department of Medicine, University of California, San Francisco, CA, USA; 2 Dept of Biostatistics, University of Florida, Gainesville, FL, USA; 3 Dept of Epidemiology, Johns Hopkins Bloomberg School of Public Health, Baltimore, Maryland, USA

## Abstract

Reliable assessment of antimalarial drug efficacy is crucial for effective response to emerging drug resistance, and therapeutic efficacy studies (TES) are the primary means of estimating *in vivo* efficacy. Accuracy of such estimates rests on correctly classifying recurrent infections developed during follow-up as recrudescences or new infections. Genotyping is used to guide classification, but polyclonal infections and allele chance matching still make classification challenging, especially in high transmission settings. Established methods for analyzing genotyping data are biased or difficult to use; few take full advantage of data from modern genotyping methods such as multiplexed amplicon sequencing. We propose an Adaptable Statistical framework for Therapeutic Efficacy and Recrudescence (Aster) that delivers accurate and consistent results by explicitly incorporating complexity of infection (COI), population allele frequencies, and imperfect detection of alleles in minority strains. Using an identity by descent approach, Aster accounts for alleles matching by chance and background relatedness that can otherwise lead to misclassification. The extensible framework can also utilize external information, such as parasite density and performance characteristics of a genotyping panel. Aster employs efficient combinatorial algorithms to process unphased polyclonal data, making it fast and fully scalable. Using simulations, we show that Aster dramatically outperforms match-counting algorithms currently recommended by WHO in a wide variety of settings and demonstrates consistently balanced performance measures that improve with more informative genotyping panels. Aster provides accurate study-level estimates of treatment failure for TES with any type of genotyping data, facilitating reliable evaluation of drug efficacy and effective management of malaria.

## 1 Introduction

Widespread availability of highly effective artemisinin combination therapies (ACTs) has greatly improved treatment for *Plasmodium falciparum* infection, the most fatal cause of malaria, and has contributed to a global reduction in malaria burden over the last 2 decades. Unfortunately, progress in malaria control has recently stalled, with over 600,000 malaria deaths estimated in 2023 [1]. One of the largest threats to malaria control right now is the emergence of artemisinin partial resistance in sub-Saharan Africa, where > 95% of malaria cases and deaths occur [2, 3]. Treatment with a failing drug often results in partial clearance of infecting parasites, which then recrudesce, resulting in increased morbidity, mortality, and ongoing transmission of resistant parasites. Antimalarial efficacy is primarily evaluated through therapeutic efficacy studies (TES). A TES assesses *in vivo* efficacy by enrolling patients with symptomatic malaria infections, administering directly observed therapy, and actively following these individuals to assess parasite clearance. Since recrudescence often occurs several weeks after therapy, extended follow-up times are required to assess for recurrent parasitemia. Importantly, the World Health Organization (WHO) recommends changing therapy if genotype-corrected failure (recrudescence) rates exceed 10%, making TES results a vital source of information to determine treatment policy.

In malaria endemic areas, TES participants are often reinfected during the long follow-up period. Measuring efficacy thus requires distinguishing whether detection of asexual parasites during follow-up is due to a recrudescence, suggesting true drug failure, or a new infection, which is not an indication of drug failure [4, 5]. In areas with high malaria transmission, including most of sub-Saharan Africa where artemisinin partial resistance is now emerging, people can be infected hundreds of times per year, and over 50% of study participants can develop new infections during follow-up [6, 7]. Results of TES in these areas – and by extension treatment policy – can therefore be dramatically affected by the accuracy of outcome classification. The classification is accomplished by comparing the genotypes of parasites present in the blood prior to treatment (day zero [D0]) with those present when recurrent parasitemia is first detected by microscopy (day of recurrence [DR]). Since *P. falciparum* populations in sub-Saharan Africa exhibit high levels of genetic diversity, it is unlikely that an individual will, by chance, be reinfected with a genetically identical parasite. If parasite genotypes from DR match those on D0, then recurrent parasitemia is likely due to a recrudescence; if they do not match, then it is likely due to a new infection [5].

In practice, biological and technical factors complicate interpretation, compromising the accuracy of TES results [3, 8]. *P. falciparum* infections frequently contain multiple genetically distinct strains, i.e. have a complexity of infection (COI) greater than 1 [9, 10]. While genotyping can usually detect multiple alleles at each locus, it is not currently feasible to assign alleles across loci to underlying strains (i.e. the genotyping data are unphased). Since a recurrent infection would constitute a recrudescence event if it contains at least one recrudescent strain, regardless of the presence or absence of newly infecting strains, any matching alleles between D0 and DR at a given locus provide potential evidence of recrudescence. However, alleles from genetically distinct parasites can also match by chance, potentially resulting in misclassifying a new infection (i.e. an infection with no original pre-treatment strains) as a recrudescence; polyclonality exacerbates the issue by contributing to chance matching. To decrease the probability of this misclassification, the current practice recommended by the WHO is to genotype 3 diverse loci, assuming that matches at all 3 would indicate recrudescence [11]. Technical issues pose additional challenges for classification: misidentification of genotyping artifacts as additional alleles (i.e. false positive alleles) can produce non-existent matches, while alleles from strains at low frequencies in the blood might go undetected (i.e. false negative alleles), potentially precluding identification of recrudescence. These and other factors can result in misclassification of outcomes and biased TES results [12, 13].

There is no consensus on how genotyping data should be analyzed to classify TES outcomes. The method currently recommended by the WHO is a match-counting approach where genotypes from D0 and DR are considered a recrudescence if they have at least one matching allele at all 3 of the loci (“3/3 algorithm”) [14]. To allow for imperfect sensitivity of genotyping methods, the “2/3 algorithm” has been proposed as an alternative, requiring only 2 of the 3 loci to have matching alleles. Both approaches are easy to implement, but do not explicitly account for factors that can have a large effect on the presence of matching alleles outside of recrudescence, such as allele frequency, COI, and background relatedness. The end result is estimates of drug failure that may be considerably biased by factors having nothing to do with the risk of drug failure, such as transmission intensity or the genotyping method used [12]. A more recent approach, the CDC microsatellite algorithm, is a Bayesian model that explicitly accounts for several of these factors [15, 12]. However, it is not currently recommended by the WHO due to its lengthy run times; further, this method is designed for length polymorphisms and not tailored to amplicon sequencing methods which may have advantages in accuracy, accessibility, timing, and cost [16, 17, 18, 19]. The consequence is that TES results are often reported using biased, inconsistently applied analysis methods [3, 8, 13].

Here, we propose a novel statistical approach, Adaptive Statistical framework for Therapeutic Efficacy and Recrudescence (Aster) that performs recurrence classification when comparing genotyping data from pairs of samples from trial participants with recurrent infections. This flexible framework is efficient and fully scalable to any number of loci and alleles. We hypothesized that implementing a version of Aster accounting for allele frequency of parasites, COI and background relatedness of infections, and imperfect allele detection would provide more consistent and accurate results than match-counting algorithms across a variety of genotyping methods and epidemiologic settings. First, we present the overall framework, then evaluate this approach using a range of simulated data sets, and conclude with discussing its practical applications.

## 2 Methods

### 2.1 Framework

Aster is designed to infer therapeutic failure rates for antimalarial TES. The adaptive framework can be described most generally as consisting of two main components: the genetic data generating model and the detection model. We assume independence between the corresponding random processes. Each of the models can be adapted to different settings and can utilize external information allowing for flexibility in practical applications. Overlaying these models is an observation mechanism, which reflects the fact that phased genotype data are not usually directly observed; this mechanism can be formulated as a deterministic function. At the top level, the framework addresses recrudescence, allowing both strain and person-specific definitions. Here we present the latter, most common, approach where recrudescence is defined as an event where at least one strain from D0 infection is recrudescent. In this case, therapeutic failure rate is the probability that an individual has a recrudescence event following therapy. First, we describe the framework components and observation mechanism for a single sample, not considering recrudescence, then expand them to a pair of samples from the same individual. The relationship between recrudescence and identity by descent (IBD) is a part of the genetic model and is described in Section 2.1.2.

#### 2.1.1 Single sample

##### Genetic data generating model

**Figure 1:**
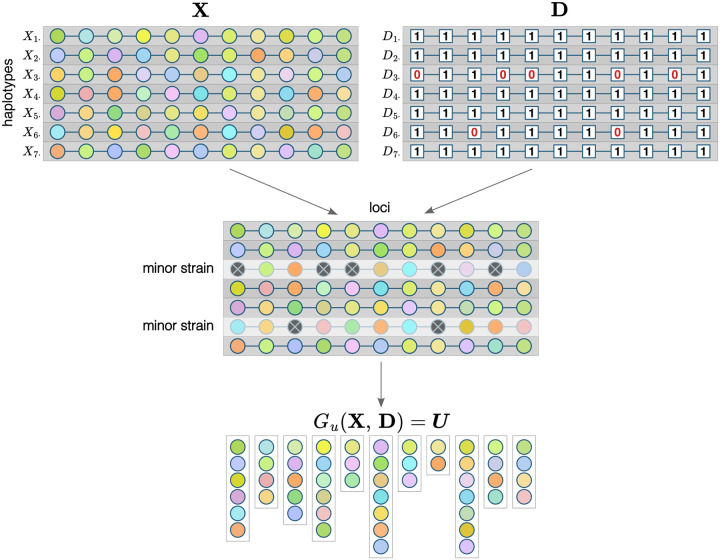
Framework components for a single sample. Each row of X and D matrices represents a strain, each column - a locus. Colors inside circles represent alleles; multiple strains can have the same allele at a given locus. Minor strains are not fully detected, which is indicated with crossed out dark gray circles, but an allele that is undetected in one strain can still be detected at a locus if it is present in another strain. Observed data for each locus is a set of detected unique alleles.

Let X be a n×L matrix of random variables, where n is COI, L is the number of loci, rows $X_{i\cdot }=\left(X_{i1},\ldots ,X_{iL}\right)$ represent distinct parasite genotypes, and columns $X_{\cdot l}=\left(X_{1l},\ldots ,X_{nl}\right)$ represent alleles at each locus (Figure 1). X can be thought of as phased genotype data for a single infection. Each $X_{il}$ has a categorical distribution with values in a set $A_{l}=\left\{a_{l1},\ldots ,a_{lK_{l}}\right\}$ of possible alleles at locus l and corresponding probabilities - population allele frequencies $\pi \left(a_{lk}\right)$. $X_{il}$ are assumed to be independent, which implies no linkage disequilibrium, perfect mixing of parasite strains, and no intrahost relatedness.


$$P(X)=\prod_{i=1}^{n}\prod_{l=1}^{L}\pi \left(X_{il}\right)$$

##### Detection model

In a polyclonal infection, parasite strains that are present in a sample in smaller proportions (minor strains) might be harder to detect during genotyping, and for these strains, alleles at some loci might not be detected. Let D be a binary n×L matrix that represents detection: if $D_{il}=1$, an allele in strain $X_{i\cdot }$ at locus l has been detected, and if $D_{il}=0$, it has not been (Figure 1). $D_{il}$ are independent random variables, $D_{il}\sim \text{Bernoulli}\left(p_{il}\right)$, where $p_{il}$ is a probability of detection. In addition to strain proportion, $p_{il}$ can depend on panel characteristics, overall parasite density, COI, and other factors. In many cases, it is reasonable to assume the same probability for alleles from a strain to be detected at any locus $\left(p_{i1}=\ldots =p_{iL}\right)$, but it is possible that some markers can be more sensitive, in which case these probabilities will vary from locus to locus. Major strains can be assumed to be fully detected ($p_{jl}=1\forall l$ for some major strain j).


$$P(D)=\prod_{i=1}^{n}\prod_{l=1}^{L}p_{il}^{D_{il}}{\left(1-p_{il}\right)}^{1-D_{il}}$$

##### Observation mechanism

When genetic data for polyclonal infections are unphased, observed data consist of unordered lists of unique alleles detected at each locus. Define a function $\left.g_{u}\left({\left(a_{i}\right)}_{i=1}^{n},{\left(b_{i}\right)}_{i=1}^{n}\right)\right)$ that maps two sequences of the same length, the second of which is binary, to a set $U=\left\{a_{i}:b_{i}=1\right\}$. Applying this function to columns of X and D, we get a sequence of sets, $U=\left(U_{1},\ldots ,U_{L}\right)=\left(g_{u}\left(X_{\cdot 1},D_{\cdot 1}\right),\ldots ,g_{u}\left(X_{\cdot L},D_{\cdot L}\right)\right)$ that represents our observed data. For convenience, let $G_{u}(X,D)\equiv {\left(g_{u}\left(X_{\cdot l},D_{\cdot l}\right)\right)}_{l=1}^{L}$ denote a deterministic function representing the observation mechanism for a whole sample. Note that if an allele from a minor strain i at locus l has not been detected ($D_{il}=0$), it can still belong to $U_{l}$ if it is present and has been detected in other strains (Figure 1).

##### Probability of observed genotyping data

Since $X_{il}⊥D_{jm}\forall i,j,l,m$, the probability of observed data U for an infection with n distinct clones can be written simply as

(1) $$P(U)=\sum_{X,D:\;G_{u}(X,D)=U}P(X)P(D)$$

The summation in Equation (1) is taken over all the combinations of full genetic and detection matrices that are compatible with observed data, thus making this direct approach computationally unfeasible for most cases and generally unscalable; including a second sample combined with recrudescence adds another layer of complexity. While Aster uses other approaches described below to efficiently calculate the likelihood, it is a useful reference to what exactly needs to be calculated. Equation (1) does not explicitly exclude matrices with repeated rows, and there is a non-zero probability that two unrelated strains are identical by state at all the loci in a panel. However, if the panel is informative (i.e. has a sufficient number of diverse loci), this probability is small; in addition, if $\left|U_{l}\right|=n$ for at least one locus l, which is often the case in practice as most COI estimation methods for multiallelic data would yield estimates that are no greater than $\text{max}\left(\left|U_{1}\right|,\ldots ,\left|U_{L}\right|\right)$, no matrices with repeated rows will be compatible with U.

Using a single sample, we introduce some of the concepts of Aster implementation. First, since loci can be considered somewhat separately (keeping in mind that for both recrudescence and detection, strains/rows need to be considered as a whole, with their indices fixed across loci), we can switch the order of the sum and the product, greatly reducing the number of combinations:

$$P(U)=\sum_{\begin{array}{c} X,D:\\ G_{u}(X,D)=U \end{array}}P(X)P(D)=\sum_{\begin{array}{c} X,D:\\ G_{u}(X,D)=U \end{array}}\left[\prod_{l=1}^{L}P\left(X_{\cdot l}\right)\right]\;\left[\prod_{l=1}^{L}P\left(D_{\cdot l}\right)\right]=\prod_{l=1}^{L}\sum_{\begin{array}{c} X_{\cdot l},D_{\cdot l}:\\ g_{u}\left(X_{\cdot l},D_{\cdot l}\right)=U_{l} \end{array}}P\left(X_{\cdot l}\right)P\left(D_{\cdot l}\right)$$

Next, we define a function that allows us to calculate a probability of a set of unique alleles at a given locus without explicitly allocating these alleles to specific strains. Let $P_{u}(U,n)$ be a function that calculates a probability that a sequence of length n has all the elements of set U with corresponding probabilities and only elements from set U: for a sequence $Z_{1},\ldots ,Z_{n}$ of independent identically distributed categorical random variables,

(2) $$P_{u}(U,n)=P\left(Z_{i}\in U\forall i\;\text{and}\;\forall a\in U\exists j:Z_{j}=a\right).$$

Set $P_{u}(\emptyset ,0)=1$; also note that $P_{u}(U,n)=0$ if |U|>n. In Aster, elements of U are alleles, their corresponding probabilities are population allele frequencies, and n is the number of strains to which alleles in U belong. Supplementary section S.1 provides two efficient ways to calculate $P_{u}(U,n)$.

To incorporate missingness, recall that $P\left(D_{il}=1\right)=p_{il}$. If $D_{il}=0,\;X_{il}$ is unknown and can be any allele $a\in A_{l}$; consequently, if alleles from all the other strains j≠i are detected, $P\left(U_{l}|n,D_{il}=0\right)=P_{u}\left(U_{l},n-1\right)$ (see Supplementary Section S.2 for details). Then, for a sample with COI of n and a single minor strain i,

(3) $$P\left(U_{l}|n\right)=P\left(U_{l}|n,D_{il}=1\right)\;P\left(D_{il}=1\right)+P\left(U_{l}|n,D_{il}=0\right)\;P\left(D_{il}=0\right)\;\;=p_{il}P_{u}\left(U_{l},n\right)+\left(1-p_{il}\right)\;P_{u}\left(U_{l},n-1\right)$$

for locus l.

In general, for any set of minor strains,

(4) $$P\left(U_{l}|n\right)=\sum_{D_{\cdot l}\in {ℬ}^{\left(n\right)}}\left[\prod_{i:\;D_{il}=1}p_{il}\right]\;\left[\prod_{j:{\;D}_{jl}=0}\left(1-p_{jl}\right)\right]P_{u}\left(U_{l},\sum_{i=1}^{n}D_{il}\right)$$

where ${ℬ}^{\left(n\right)}$ is a set of all the possible binary sequences of length n (note that the terms with $D_{\cdot l}$ such that $\sum_{i=1}^{n}D_{il}<\left|U_{l}\right|$ are equal to 0).

#### 2.1.2 Pair of samples and recrudescence

Recrudescence can be viewed at a person level (an individual in a study) or, alternatively, considering that many infections are polyclonal, at a strain level. Commonly, when therapeutic failure rates are estimated, a failure is defined on a person level - even if multiple strains in a single infection have recrudesced. Adopting this approach, we consider a case where at least one of the D0 strains has not completely cleared and is present in the DR sample (regardless of the presence or absence of newly accquired strains) to be a recrudescence event. Let $R_{j}$ be a binary random variable representing recrudescence for individual j where $R_{j}=1$ indicates a recrudescence event, and let θ be a population-level failure rate. In TES, individual responses to therapy are commonly considered to be independent, so we assume $R_{j}\sim \text{Bernoulli}(\theta )$, j=1,…,N, where N is the number of individuals in the study, to be independent and identically distributed.

##### Genetic data generating model

Let X and Y be $n_{x}\times L$ and $n_{y}\times L$ matrices representing D0 and DR infections from the same individual. We assume that newly infecting clones are acquired independently from recrudescence, and consequently that recurrence of parasitemia can be a result of a new infection, a recrudescence, or both. If Y has recrudescent strains, the rows representing them are identical to the corresponding rows in X, while entries $Y_{jl}$ in the rows of Y that represent newly infecting strains (if any) are drawn from the same distribution as $X_{il}$. Since the order of the strains within an infection does not matter, we order them in such a way that recrudescent strains occupy the top rows of both matrices and their indices match in X and Y, e.g. if an individual has two recrudescent strains, then $X_{1l}=Y_{1l}\forall l$ and $X_{2l}=Y_{2l}\forall l$.

We approach modeling alleles in recrudescent strains by considering identity by descent. A recrudescent strain i is the same in both samples, and it can be stated that $X_{il}$ and $Y_{il}$ are IBD at all loci. Let $IBD_{il}$ be an indicator that $X_{il}$ and $Y_{il}$ are IBD. Then

$$P\left(X_{il},Y_{il}|IBD_{il}=1\right)=\left\{\begin{array}{ll} \pi \left(X_{il}\right) & \text{if}\;X_{il}=Y_{il}\\ 0 & \text{otherwise} \end{array}\right.$$

$$P\left(X_{il},Y_{il}|IBD_{il}=0\right)=\pi \left(X_{il}\right)\;\pi \left(Y_{il}\right)$$

For a pair of strains $X_{i\cdot },\;Y_{i\cdot }$, let $R_{i}^{\prime }$ be an indicator that strain i is recrudescent (we want to distinguish a strain specific variable $R_{i}^{\prime }$ from R, a person-level recrudescence). If $R_{i}^{\prime }=1,\;IBD_{il}=1\;\forall l$; for the reverse to be true, we would need to assume that a person cannot be reinfected with strains that are related to the ones present at D0. That is not necessarily the case, and a population can have a non-zero level of background relatedness $r_{bg}$, which we define as a probability that a newly infecting strain and an originally present strain are IBD at a given locus. Then

$$P\left(IBD_{il}=1|R_{i}^{\prime }=1\right)=1$$

$$P\left(IBD_{il}=1|R_{i}^{\prime }=0\right)=r_{bg}$$

##### Detection model

Let $D_{x}$ and $D_{y}$ be $n_{x}\times L$ and $n_{y}\times L$ binary matrices representing detection for X and Y, with corresponding probabilities of detection $P\left(D_{x,il}=1\right)=p_{x,il}$ and $P\left(D_{y,il}=1\right)=p_{y,il}$. In addition to independence between full haplotype data and detection, we assume independence between detection in two samples $D_{x,il}⊥D_{y,jm}\forall \;i,j,l,m$ as well as independence between detection and recrudescence/IBD status: $D_{x,il}⊥IBD_{il}$ and $D_{y,il}⊥IBD_{il}$.

##### Observation mechanism

We denote observed data for samples from D0 and DR by $U_{x}=\left(U_{x,1},\ldots ,U_{x,L}\right)=G_{u}\left(X,D_{x}\right)$ and $U_{y}=\left(U_{y,1},\ldots ,U_{y,L}\right)=G_{u}\left(Y,D_{y}\right)$ respectively.

##### Likelihood

In general,

(5) $$L\left(\theta ;U_{x},U_{y}\right)=\theta \;P\left(U_{x},U_{y}|R=1\right)+(1-\theta )\;P\left(U_{x},U_{y}|R=0\right)$$

$P\left(U_{x},U_{y}|R=0\right)=P\left(U_{x}\right)\;P\left(U_{y}\right)$, but to derive $P\left(U_{x},U_{y}|R=1\right)$, we need to consider strain-level recrudescence and look at probabilities with given sets of indices for minor and recrudescent strains (recall that recrudescent strain indices should match in X and Y). We start with a base case example where a possibly recrudescent strain is also a minor strain in both samples, then proceed to the general case; for derivations and special cases, see Supplementary Section S.2. Suppose there is a single strain i=1 that might be recrudescent, a single minor strain $X_{1\cdot }$ in the D0 sample and a single minor strain $Y_{1\cdot }$ in the DR sample; also assume that strains $Y_{k\cdot },\;k>1$, if any, are not related to any strains in the D0 sample. Then

$$P\left(U_{x},U_{y}\right)=\theta \prod_{l=1}^{L}P\left(U_{x,l},U_{y,l}|IBD_{1l}=1\right)\;\;+(1-\theta )\prod_{l=1}^{L}\left[P\left(U_{x,l},U_{y,l}|IBD_{1l}=1\right)r_{bg}+P\left(U_{x,l},U_{y,l}|IBD_{1l}=0\right)\left(1-r_{bg}\right)\right]$$

(see Supplementary Section S.3).

To find $P\left(U_{x,l},U_{y,l}|IBD_{1l}\right)$, we need to account for all four combinations of $\left(D_{x,1l},D_{y,1l}\right)$ and find $P\left(U_{x,l},U_{y,l}|D_{x,1l},D_{y,1l},IBD_{1l}\right)$:

$$P\left(U_{x,l},U_{y,l}|n_{x},n_{y},IBD_{1l}=1\right)={\;p}_{x,1l}\;p_{y,1l}P_{xy}^{\left(1\right)}\left(U_{x,l},U_{y,l},n_{x},n_{y}\right)\;+p_{x,1l}\left(1-p_{y,1l}\right)P_{u}\left(U_{x,l},n_{x}\right)\;P_{u}\left(U_{y,l},n_{y}-1\right)\;+\left(1-p_{x,1l}\right)\;p_{y,1l}\;P_{u}\left(U_{x,l},n_{x}-1\right)\;P_{u}\left(U_{y,l},n_{y}\right)\;+\left(1-p_{x,1l}\right)\left(1-p_{y,1l}\right)P_{u}\left(U_{x,l},n_{x}-1\right)\;P_{u}\left(U_{y,l},n_{y}-1\right),$$

where

(6) $$P_{xy}^{\left(1\right)}\left(U_{x},U_{y},n_{x},n_{y}\right)=\sum_{a\in U_{xy}}\pi (a)\left[P_{u}\left(U_{x},n_{x}-1\right)+P_{u}\left(U_{x}\setminus \{a\},n_{x}-1\right)\right]\times \left[P_{u}\left(U_{y},n_{y}-1\right)+P_{u}\left(U_{y}\setminus \{a\},n_{y}-1\right)\right],$$

$U_{xy}=U_{x}\cap U_{y}$, and a set difference U∖{a} is a set U without an element $a.\;P_{xy}^{\left(1\right)}\left(U_{x},U_{y},n_{x},n_{y}\right)$ is a function that accounts for a single recrudescent strain between two samples using Equation (2). When $IBD_{1l}=0$,

$$P\left(U_{x,l},U_{y,l}|n_{x},n_{y},IBD_{1l}=0\right)=P\left(U_{x,l}|n_{x}\right)\;P\left(U_{y,l}|n_{y}\right),$$

where $P\left(U_{x,l}|n_{x}\right)$ and $P\left(U_{y,l}|n_{y}\right)$ are calculated as in Equation (3).

In general, to accommodate any number and combination of possibly recrudescent strains, IBD pairs of strains, and minor strains, whether recrudescent or not, in both samples, we combine Equation (4) with the generalized conditional probability, which is the key component of the likelihood:

$$P\left(U_{x,l},U_{y,l}|D_{x,\cdot l},D_{y,\cdot l},IBD_{\cdot l}\right)=P_{xy}^{\left(m\right)}\left(U_{x,l},U_{y,l},\sum_{i=1}^{n_{x}}D_{x,il},\sum_{j=1}^{n_{y}}D_{y,jl}\right)\;,$$

where $IBD_{\cdot l}$ is a sequence ${\left(IBD_{il}\right)}_{i=1}^{min\left(n_{x},n_{y}\right)},\;m=\sum_{i=1}^{min\left(n_{x},n_{y}\right)}D_{x,il}D_{y,il}IBD_{il}$, and

(7) $$P_{xy}^{\left(m\right)}\left(U_{x,l},U_{y,l},n_{x},n_{y}\right)=\sum_{U^{\prime }\subseteq U_{xy}}P_{u}\left(U^{\prime },m\right)\sum_{U^{\prime \prime }\subseteq U^{\prime }}P_{u}\left(U_{x,l}\setminus U^{\prime \prime },n_{x}-m\right)\sum_{U^{\prime \prime \prime }\subseteq U^{\prime }}P_{u}\left(U_{y,l}\setminus U^{\prime \prime \prime },n_{y}-m\right)$$

Terms with $\left|U^{\prime }\right|>m$ in Equation (7) are equal to 0 since P(U,n)=0 for |U|>n. Note that

(8) $$P_{xy}^{\left(0\right)}\left(U_{x,l},U_{y,l},n_{x},n_{y}\right)=P_{u}\left(U_{x,l},n_{x}\right){\;P}_{u}\left(U_{y,l},n_{y}\right).$$

Alternatively, multisets can be used to calculate $P\left(U_{x,l},U_{y,l}|D_{x,\cdot l},D_{y,\cdot l},IBD_{\cdot l}\right)$; this approach can be more efficient in some situations. A mixed radix system algorithm [20, 21] is used for implementation of both approaches.

Equation (8) provides guidance for the treatment of missing data (no alleles detected at some loci in one or both samples). The proof that excluding such loci from the analysis does not introduce any bias can be found in Supplementary Section S.4.

#### 2.1.3 Adaptivity

The framework can incorporate different types of available information and can be combined with other methods. Depending on the amount of external information and prior knowledge, additional assumptions and constraints can be used. Finally, it can accommodate different parameters of interest and produce a corresponding output, e.g. strain-level recrudescence.

Aster explicitly accounts for population allele frequencies and COI. Allele frequencies can be estimated from D0 samples, from a combination of D0 and new infections in recurrent samples, or can be provided from larger sample estimates with information borrowed from other studies; COI can be estimated using genetic data from a single sample or larger datasets. Similarly, background relatedness can be estimated from D0 samples or its distribution can be estimated from other sources; other information can be available to estimate it for a particular individual. The detection model has a flexible number of parameters, where probability of detection can vary between samples, loci, and strains. They can depend on sample parasite density, COI, and characterics of the genotyping panels such as sensitivity and diversity of the markers. Prior information on therapeutic failure rates can be accommodated (and might differ for different drug combinations) with a Bayesian approach; different treatments could be compared using different priors.

Instead of a fixed value or point estimate, a distribution of $r_{bg}$ might be available and can be incorporated into analysis. Let $f_{bg}(r)$ be a probability density function (pdf) of $r_{bg}$. Then

$$L\left(\theta ;U_{x},U_{y}\right)=\int_{0}^{1}L\left(\theta ;U_{x},U_{y},r_{bg}\right)f_{bg}\left(r_{bg}\right)dr_{bg}$$

Similarly, distributions of detection probabilities can be incorporated. As an example, let the D0 sample have a single minor strain i with $p_{x,i1}=\cdots =p_{x,iL}=p_{x}$ and the DR sample have a minor strain j with $p_{y,j1}=\cdots =p_{y,jL}=p_{y}$; let $f_{x}(p)$ and $f_{y}(p)$ be pdf’s of $p_{x}$ and $p_{y}$ respectively. Then

$$L\left(\theta ;U_{x},U_{y}\right)=\int_{0}^{1}\int_{0}^{1}L\left(\theta ;U_{x},U_{y},p_{x},p_{y}\right)\;f_{x}\left(p_{x}\right)\;f_{y}\left(p_{y}\right)\;dp_{y}\;dp_{x}$$

Since true recrudescence status for recurrent samples is inferred and not known for certain, there are no simple conjugate priors for the Bayesian approach. However, the posterior distribution of θ can be calculated numerically:

(9) $$f_{\theta |U_{x},U_{y}}(s)=\frac{L\left(s;U_{x},U_{y}\right)f_{\theta }(s)}{\int_{0}^{1}L\left(u;U_{x},U_{y}\right)f_{\theta }(u)du},$$

where $f_{\theta }(u)$ and $f_{\theta |U_{x},U_{y}}(u)$ are prior and posterior pdf’s of θ.

### 2.2 Classification and inference

To classify a recurrence as a recrudescence or a new infection, we use the likelihood in Equation (5). The likelihood is linear, and classification can be based on the sign of the slope of the likelihood, or, in other words, on comparing $P\left(U_{x},U_{y}|R=1\right)$ and $P\left(U_{x},U_{y}|R=0\right)$. Formally, for a person-level recrudescence,

$$P\left(U_{x},U_{y}|R=1\right)=P\left(U_{x},U_{y}|M=1\right)P(M=1)+\cdots +P\left(U_{x},U_{y}|M=n_{xy}\right)P\left(M=n_{xy}\right)$$

where $n_{xy}=\text{min}\left(n_{x},n_{y}\right)$ and $M=\sum_{i=1}^{n_{xy}}R_{i}^{\prime }$ is the number of recrudescent strains. In most cases, however, it is sufficient to only consider a case with M=1: if multiple strains are recrudescent while M=1 is assumed, this only strengthens the evidence that R=1, and if there is more evidence for M=0 than for M=1, it is unlikely that multiple recrudescent strains are present. While it is theoretically possible for the slopes of the likelihood functions with and without M=1 assumption to have different signs (e.g. if P(M=1)≪P(M>1)), such a case is unlikely to represent a realistic scenario.

For classification inference, a useful measure would quantify the uncertainty of the individual classification, for which a posterior probability $P\left(R=1|U_{x},U_{y}\right)$ is a natural choice [15]. Let $A_{1}\equiv P\left(U_{x},U_{y}|R=1\right)$ and $A_{0}\equiv P\left(U_{x},U_{y}|R=0\right)$. Then

$$P\left(R=1|U_{x},U_{y}\right)=\frac{A_{1}P(R=1)}{A_{1}P(R=1)+A_{0}P(R=0)}$$

Using an empirical Bayes approach, we borrow information from all the individuals in the study by first estimating 1-θ with Kaplan-Meier survival estimator $\hat{S}\left(t_{\text{end}}\right)$ [22], where $t_{\text{end}}$ is the end of the follow-up period, and then using that estimate as a prior probability:

$$P\left(R=1|U_{x},U_{y}\right)=\frac{A_{1}\left(1-\hat{S}\left(t_{\text{end}}\right)\right)}{A_{1}\left(1-\hat{S}\left(t_{\text{end}}\right)\right)+A_{0}\hat{S}\left(t_{\text{end}}\right)}$$

In effect, this approach provides a formal way to compare $A_{0}$ and $A_{1}$, weighted by prior probabilities. If we assume a prior probability $P(R=1)=0.5,\;P\left(R=1|U_{x},U_{y}\right)=A_{1}/\left(A_{1}+A_{0}\right)$.

In addition, available external information that would contribute to prior probability or distribution can be similarly incorporated (e.g. as shown in Equation (9)).

### 2.3 Simulations

We used simulations to compare performances of Aster and match-counting algorithms for various genotyping panels, transmission intensities, genotyping error scenarios, and background relatedness levels. Panel comparisons were based on the set of 3 length polymorphisms (LP) currently recommended by the WHO (*msp1*, *msp2*, and a single microsatellite) [14], and a contemporary amplicon sequencing panel containing diverse microhaplotypes (MAD^4^HatTeR) [17]. For the amplicon sequencing panel, subsets of loci for simulations were chosen based on their heterozygosity ranking using population allele frequencies obtained from the previously analyzed datasets. To reflect higher genotyping error rates of LP panels, we increased missingness (1 - probability of detection) twice and false positive rates 5 times compared to the amplicon sequencing panels. Transmission intensity levels were reflected in sample COI and the number of new infections where applicable. COI were drawn from zero-truncated Poisson (ZTP) distributions, with D0 means of 1.2, 3, and 5 for low, moderate, and high transmission settings respectively; DR means were respectively decreased by a factor of 1.8 or set to 1, whichever was greater. Genotyping errors were included with a simple “per strain” detection model for missing (false negative) alleles and a “split” model for false positive alleles ([20]).

We used three types of simulation schemes with increasing complexity: simulated pairs of samples to evaluate classification performance, simulated studies with fixed proportions of recrudescences and newly acquired infections, and studies with simulated responses to treatment combinations using an established pharmacokinetic/pharmacodynamic (PK/PD) model [23, 24, 25]. With the first simulation scheme, groups of three samples were generated: a D0 sample and two DR samples, with and without recrudescence. We simulated 10,000 such triads for each combination of transmission intensity, allele detection probability, background relatedness, and genotyping panel. With the second scheme, 1000 studies were simulated for each combination. While the proportions of individuals with recrudescent strains and with newly infecting ones were fixed to remove variability due to the randomness of recrudescence events, the individuals to whom these strains were assigned were random, creating a random number of overlaps; thus the numbers of total recurrences varied as well. The number of recrudescent strains for each individual was drawn from a ZTP distribution with a mean of 1/3 of their D0 COI. For PK/PD simulations, parasite density profiles were generated using model parameters suggested in [25] with adjusted half-maximal inhibitory concentrations and maximum kill rates to achieve therapeutic failure rates at or near 0.1 [26, 27]. We used homogeneous Poisson processes to introduce new infection times with incidence per year of 6 for moderate and 12 for high transmission; the numbers of coinfecting strains were drawn from ZTP distributions with means dependent on transmission intensity (2.5 for moderate, 4 for high transmission. To mimic a TES protocol, we measured parasite density at regular time points; once a detection limit of 10^8^ parasites/body had been reached, a recurrence was confirmed.

Where applicable, we used naive COI estimation with a locus rank and a probabilistic COI-adjusted method ([20]) to estimate population allele frequencies for Aster. Allele detection probabilities in PK/PD simulations were estimated from sample parasite density using empirical data from mixedstrain controls for model fitting. While false positive alleles are not explicitly included in the Aster framework, its implementation allows for processing genetic data with false positives. When the number of alleles detected at a locus is greater than or equal to the COI, we allow for a possibility that some alleles might be false positives and therefore some might still be undetected by including a tuning parameter regulating a probability of this scenario. A small value for such probability would not affect classification results unless there is overwhelming evidence of recrudescence at other loci, in which case this evidence would be weighed against that value, and recrudescence would not be ruled out. For classification using match-counting algorithms, we calculated a proportion of loci with matches to apply these algorithms to simulated data from panels with more than 3 loci. Matches in all the loci were required for recrudescence with the 3/3 algorithm and matches in at least two thirds of the loci with the 2/3 algorithm; the same principle was used for missing data.

## 3 Results

### 3.1 Classification performance

Aster uses a probabilistic approach for recurrence classification that explicitly accounts for biological and technical factors, including COI, allele frequency, background relatedness between infections, and imperfect detection of alleles. To evaluate its performance in identifying whether a DR infection is a recrudescence or a new infection, we first simulated pairs of infections with and without recrudescences. Using different genotyping panels and a range of transmission settings, allele detection probabilities, and background relatedness levels, we compared the sensitivity and specificity of Aster to those of the 3/3 and 2/3 match-counting algorithms.

**Figure 2:**
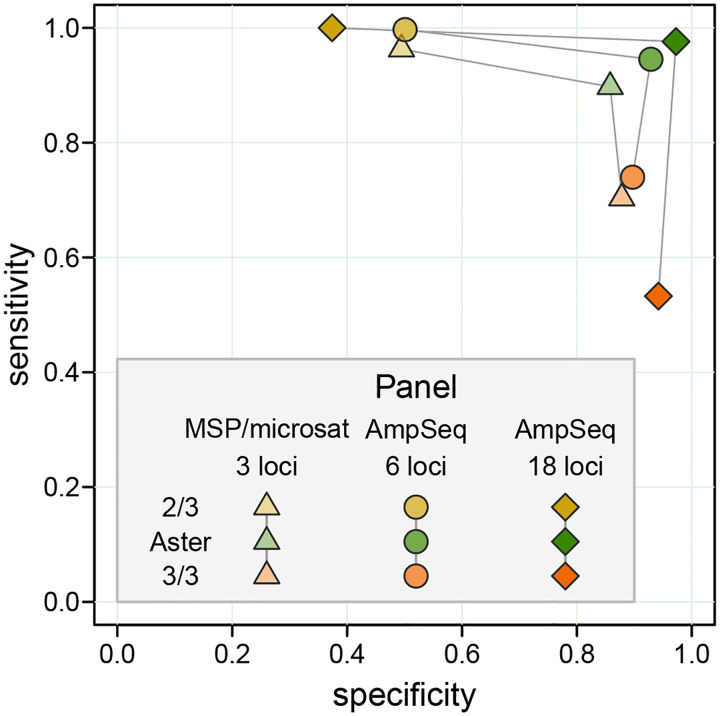
Sensitivity vs specificity for Aster, 3/3 algorithm, and 2/3 algorithm with three simulated genotyping panels: 3-locus length polymorphism (MSP/microsatellite) and 6 and 18-locus amplicon sequencing panels. A high transmission setting was used for the simulations, with a single recrudescent strain for each pair of samples and a partially detected recrudescent strain in a D0 sample; the probability of detection was set to 0.9 for all loci. For applying match-counting algorithms to panels with > 3 loci, the proportion of loci with allele matches among all loci with data in both sampes was calculated; the value of 1 was required to classify a recurrence as a recrudescence by the 3/3 algorithm and of 2/3 or greater by the 2/3 algorithm.

In a simulated high transmission setting, Aster demonstrated balanced performance, exceeding 0.85 specificity and 0.9 sensitivity for all simulated genotyping panels and reaching near perfect classification with an 18-microhaplotype amplicon sequencing panel (Figure 2). In contrast, the 3/3 match-counting algorithm had high specificity but much lower sensitivity, classifying many recurrences as new infections; the opposite was true for the 2/3 algorithm. Performance of match-counting algorithms worsened with increasing numbers of loci, reflecting the limiting nature of a mechanistic identity by state approach that does not benefit from additional information.

**Figure 3:**
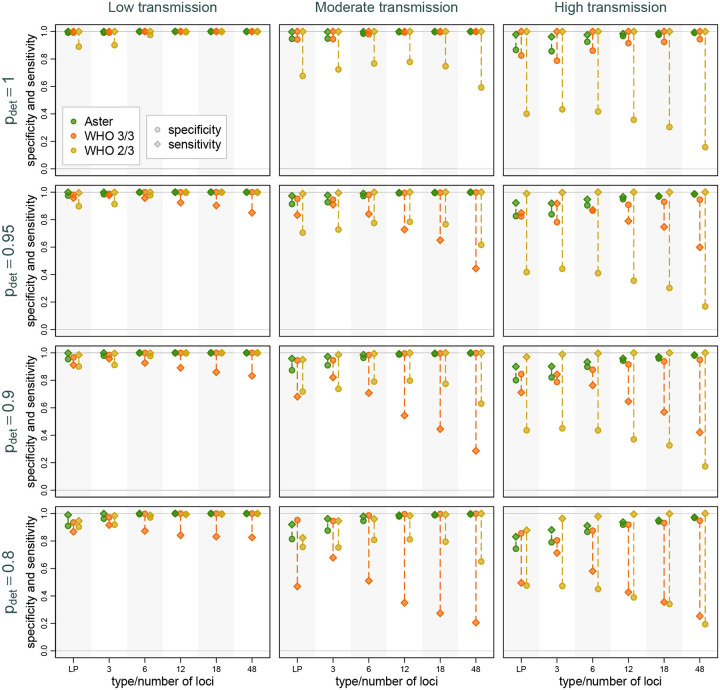
Sensitivity and specificity of Aster and match-counting algorithms across trasmission intensity levels, genotyping panels, and detection probabilities. Dotted lines mark the difference between sensitivity and specificity for a specific method in a setting; longer lines imply greater imbalance and potentially greater bias in failure rate estimation. Background relatedness was set to 0.125.

Across the full range of simulations, classification was more accurate, as expected, when alleles were more likely to be detected and when transmission was lower, due to lower COI (Figure 3). However, we found that Aster had consistently higher overall and more balanced sensitivity and specificity than either match-counting algorithm. Aster performance improved with larger, more informative panels, with 6 or more loci yielding excellent accuracy in moderate transmission and 12 or more loci in most high transmission settings. Even in simulations where recurrences were the most challenging to classify, such as high transmission settings with poor allele detection, the sensitivity and specificity of Aster remained high for the 18-locus panel and improved further with 48 loci. In contrast, while match-counting algorithms performed well in low transmission settings (with mostly monoclonal infections), the differences between sensitivity and specificity (visible as dashed lines) became pronounced at moderate and high transmission settings. This imbalance could lead to considerable overestimation or underestimation of overall failure rates, as explored below. Supplementary Figure S.1 provides similar comparisons but with varying background relatedness of infections and detection probability fixed at 0.9; as expected, higher relatedness led to decreased performance for match-counting algorithms, whereas the performance of Aster was more robust, particularly when using larger panels.

In some cases, it may be more appropriate to include a distribution of background relatedness instead of a single value (e.g. when relatedness seems overdispersed or bimodal). As a real-world example of this type of distribution, we used the empirical distribution of pairwise relatedness obtained from D0 samples from a TES recently performed in Asayita, Ethiopia (Supplementary Figure S.2). We simulated pairs of infections with background relatedness drawn from this distribution and then classified them using Aster and match-counting algorithms. Aster provided accurate and balanced results, particularly when using the more informative genotyping panels with larger numbers of diverse microhaplotype loci. In contrast, match-counting algorithms gave poor results in moderate and high transmission settings for all genotyping panels (Supplementary Figure S.3).

### 3.2 Study-level estimation accuracy

The results of a TES are interpreted at the study level, so we next simulated studies to evaluate the effect of classification accuracy on these results. First, we present the summary of the estimates obtained from studies simulated with a range of fixed proportions of individuals with recrudescent and newly infecting strains. Each study had 100 individuals, a typical size for a TES. Proportions of individuals with new strains were set to 0.1, 0.3, and 0.5 for simulated settings of low, moderate, and high transmission intensity respectively. For each combination of a genotyping panel, treatment failure rate, transmission intensity level, and probability of detection, 1000 studies were simulated, and a failure rate was estimated as a proportion of individuals with recrudescences.

**Figure 4:**
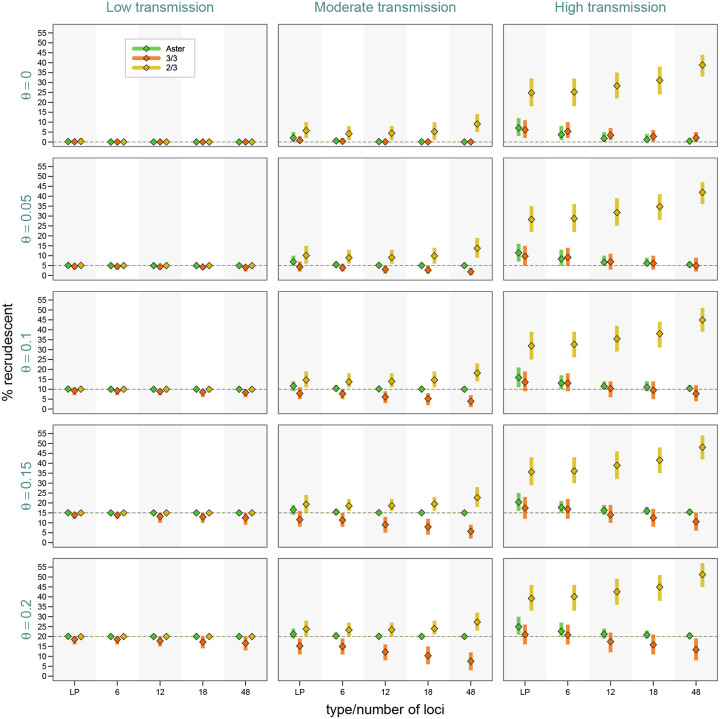
Study-level estimates across transmission intensity levels, genotyping panels, and true proportions of recrudescence events. The vertical bars represent a 95% range (0.025 to 0.975 quantiles) of the results, the diamond symbols represents a mean. Detection probability was fixed at 0.9, and background relatedness was 0.

As expected from classification results presented earlier, failure estimates based on Aster were the most accurate and increasingly concentrated around the true proportion of recrudescence with greater numbers of loci (Figure 4). For example, in the most challenging high transmission setting, when the true failure rate was 0.15, Aster produced results between 0.14 and 0.18 95% of the time with an 18-locus panel, and between 0.14 and 0.17 with a 48-locus panel. In contrast, match-counting algorithm estimates were more biased, with the extent and direction of bias depending on transmission setting, true failure rate, genotyping panel, and algorithm. In general, higher transmission resulted in greater estimate variability, more bias, and greater differences between the estimates obtained with the two algorithms; notably, more informative panels led to greater bias. The 2/3 algorithm tended to overestimate failure, with dramatic overestimation in high transmission simulations, e.g. producing estimates between 0.28 and 0.41 95% of the time for a true failure rate of 0.05 when using 18 loci. The 3/3 algorithm underestimated the failure in moderate transmission, with more biased results for higher failure rates, e.g. with 95% of the estimates between 0.06 and 0.15 for a true rate of 0.2 with an 18-locus panel; the estimates were expectedly higher for high transmission, where the bias went in either direction. To evaluate the effect of imperfect allele detection on study results, we next fixed the proportion of recrudescences at 15% and considered a range of detection probabilities. With perfect allele detection in low transmission settings, all the methods had excellent performance, which was not the case in more challenging settings. While Aster results remained close to the true values, particularly for larger microhaplotype panels, for all levels of missingness, match-counting estimates shifted downward with lower detection probabilities, as true allele matches were missed (Supplementary Figure S.4).

**Figure 5:**
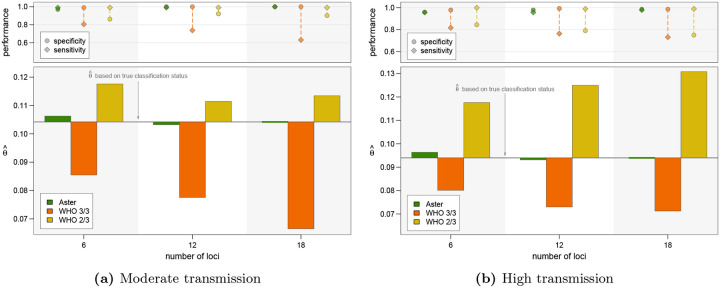
Therapeutic failure rate estimates with PK/PD model simulations using an amplicon sequencing panel with 6, 12, ro 18 loci. For each figure, the bars in the lower panel represent deviation from the estimate based on true classification status; the upper panel displays performance measures. **(a)** Simulated dataset from moderate transmission: 1000 individuals with mean COI of 3.5 for D0, incidence of 6 infections/year, and an average of 2.5 coinfecting strains per new infection. **(b)** Simulated dataset from moderate transmission: 1000 individuals with mean COI of 5 for D0, incidence of 12 infections/year, and an average of 4 coinfecting strains per new infection.

To better approximate the underlying biological and ascertainment processes of a TES, we used a previously developed PK/PD model to simulate the dynamics of parasite clones present in D0 infections, new infections acquired during the 28 day follow-up period, and the effect of antimalarial treatment with artemether-lumefantrine (Supplementary Figure S.5). For each simulated infection where recurrence would be detected, genotyping data were generated with detection based on relative and absolute parasite density (Supplementary Figure S.6). We then applied genotyping classification algorithms to these data and calculated failure rates using the Kaplan-Meier survival estimator. The results are presented for two simulations with moderate and high transmission settings respectively, each with 1000 individuals. Comparison results were qualitatively similar to the simple simulations presented above in that estimates were accurate for Aster but biased for match-counting algorithms (Figure 5). In both simulations, the 3/3 method consistently underestimated while the 2/3 method consistently overestimated failure rates. Underestimation with the 3/3 method was more pronounced in the moderate transmission setting (producing an estimate of < 0.07 using 18 loci when the true failure rate was > 0.10), and overestimation with the 2/3 method was more pronounced in the high transmission setting (producing an estimate of > 0.13 using 18 loci when the true failure rate was < 0.10). This trend is consistent with previous results and is a likely consequence of more alleles matching by chance in the high transmission setting due to higher COI.

### 3.3 Input estimation, parameter misspecification, and assumption violations

Since COI, population allele frequencies, detection probabilities, and other Aster model parameters are not usually known in practice and are estimated, inaccuracies of such estimates might affect Aster results. Therefore, we explored Aster’s sensitivity to misspecifications by testing the method with different scenarios. Estimating COI and allele frequencies from simulated data had a minimal effect on Aster results (Supplementary Figure S.7, rows 2, 4). Simulation of false positive alleles, though not formally included in the estimation process, had a minimal effect on amplicon sequencing results but did decrease accuracy for length polymorphisms, which were simulated with a higher false positive rate (Supplementary Figure S.7, rows 3, 4).

Sensitivity to misspecification of allele detection probability is of particular importance, since perfect detection is unlikely and missingness is generally difficult to estimate in practice. To assess the effect of misspecification, we simulated studies of 100 individuals and looked at the distribution of failure rate estimates obtained with different detection probabilities in a recrudescent strain in a D0 sample (Supplementary Figure S.8). Assuming perfect detection resulted in considerable underestimation when true detection probability was less than 1, but the effect of other misspecified values was much smaller. For example, at a true value of 0.7 allele detection (quite poor sensitivity), assumed values between 0.8 and 0.9 still resulted in estimates of treatment failure within 0.012 and 0.018 of the true value on average; assuming perfect detection shifted the values by nearly 0.15. Thus, in all the cases when true detection was not perfect, specifying a reasonable estimate of detection probability resulted in more accurate classification than assuming no missingness at all.

## 4 Discussion

Results from TES are critically important to inform national and regional antimalarial drug policies. For these results to be reliable, genotype-corrected outcomes should be consistently unbiased across transmission intensities and choice of genotyping assay. By explicitly accounting for population allele frequencies, COI, background relatedness, and allele detection probability within a flexible statistical framework, we found that Aster identifies recrudescent events with high sensitivity and specificity, exceeding the performance of match-counting algorithms. Improvements in accuracy were particularly salient in data simulated from high transmission areas, such as those where partial artemisinin resistance is now spreading, due to higher rates of acquiring new infections and higher COI, making outcome classification more important and more challenging. In addition, as more informative data from larger genotyping panels were included, the estimates became more accurate – a feature that will become increasingly useful as genotyping methods continue to evolve.

Unlike match-counting algorithms, Aster is able to incorporate several important factors that meaningfully impact estimates of treatment failure. The COI of infections varies with malaria transmission intensity, dramatically affecting the number of alleles matching by chance and consequently the results of traditional match-counting algorithms [9]. While the true COI of samples are unknown, they can be readily estimated from empirical data and incorporated into Aster to minimize bias in outcome classification. Similarly, allele frequencies will vary based on the loci genotyped and the local parasite population, affecting the probability of alleles matching; these too can be readily estimated from empirical data, for example using a random subset of D0 samples, and incorporated into Aster estimation. Information on background relatedness between infections is explicitly included in Aster since underestimating the relatedness in a population can lead to misclassifying a new infection as a recrudescence. As with allele frequencies, an estimate of background relatedness can be obtained from D0 samples, for example by estimating their pairwise IBD proportion, and supplied as an input to Aster, minimizing bias. Imperfect detection of alleles may also affect results but is more difficult to estimate, potentially varying with genotyping method, COI, and parasite density. Fortunately, we show that Aster is not very sensitive to misspecification of detection probability as long as detection is not assumed to be perfect. Thus, including imperfect detection in the framework calibrates missingness in a principled way, allowing Aster to correctly classify recurrence as a recrudescence when recrudescent strains are not fully detected and would likely be missed by the 3/3 algorithm. Further, while we explicitly consider genotyping errors caused by false negative (undetected) alleles, the current framework does not formally include false positive alleles. While false positives can have a deleterious effect on the accuracy of match-counting algorithms, Aster is based on an IBD approach, which takes chance matches into account, minimizing the effect of false positives on classification accuracy.

Reliable assessment of antimalarial efficacy is more pressing than ever, with resistance spreading in high transmission areas where accurate genotyping correction is the most challenging. Our results suggest that accuracy can be greatly improved through application of genotyping tools that provide rich genetic data, such as sensitive, diverse amplicon sequencing panels [17, 28], combined with analytical methods that take full advantage of these data and deliver consistent results across transmission levels. Fortunately, access to next generation sequencing has dramatically improved; over 80% of national public health labs in sub-Saharan Africa now have technology in place to generate high quality genotyping data (Africa CDC, personal communication, and [29]). Taking advantage of these data requires employing principled statistical approaches [15, 30] in place of simple matching algorithms that can provide inconsistent results which vary by genotyping panel and epidemiologic setting. Aster provides a structured statistical framework that is able to accommodate various types of information and can be amended with additional features in concordance with evolving laboratory methods and bioinformatic pipelines. With implementation that is fast and user-friendly, we show that Aster can contribute to accurate assessment of antimalarial TES, aiding stakeholders in timely determination of effective management of malaria.

## Supplementary Material

1
