# Supplementary material for: Classification of outcomes in antimalarial therapeutic efficacy studies with Aster
Source: bioRxiv. 2025 Sep 15:2025.04.28.651146. Originally published 2025 May 1. Preprint. [Version 4] doi: 10.1101/2025.04.28.651146 (PMC12247718; doi:10.1101/2025.04.28.651146)
Supplement: 1 [file NIHPP2025.04.28.651146v4-supplement-1.pdf]

bioRxiv preprint doi: https://doi.org/10.1101/2025.04.28.651146; this version posted September 15, 2025. The copyright holder for this preprint (which was not certified by peer review) is the author/funder, who has granted bioRxiv a license to display the preprint in perpetuity. It is made available under aCC-BY 4.0 International license.

Supplementary Materials

S.1 Calculation of $P_u(U, n)$

Here we describe two approaches to calculate $P_u(U, n)$ that are used in implementation of Aster:

1. For $U = \{a_1, \ldots, a_m\}$ and probabilities $\{\pi(a_1), \ldots, \pi(a_m)\}$, calculate the probability of all the sequences with elements in U, which is $\left(\sum_{i=1}^{m} \pi(a_i)\right)^n$, then subtract probabilities of the sequences that do not contain all of the elements in U using the inclusion-exclusion principal:

$$\begin{aligned} P_u(U, n) &= \left[\sum_{i=1}^{m} \pi(a_i)\right]^n - \sum_{U^{(m-1)} \subset U} \left[\sum_{a_k \in U^{(m-1)}} \pi(a_k)\right]^n + \cdots + (-1)^{m-1} \sum_{i=1}^{m} \pi(a_i)^n \\ &= \sum_{i=1}^{m} (-1)^{m-i} \sum_{U^{(i)} \subseteq U} \left[\sum_{a_k \in U^{(i)}} \pi(a_k)\right]^n \\ &= \sum_{U' \subseteq U} (-1)^{m-|U'|} \left(\sum_{a_k \in U'} \pi(a_k)\right)^n, \end{aligned}$$

where $U^{(k)}$ denotes a set of cardinality k.

2. Generate all the multisets S of cardinality n and support U: $|S| = n$, $Supp(S) = U$. $P_u(U, n)$ is the sum of all $P(S)$, which are calculated using multinomial probabilites.

The first approach is more computationally efficient for lower values of m and higher values of n. It gets slower with higher m and might encounter numerical issues (depending on a combinatorial implementation) if the orders of terms with alternating signs are much greater than the end result. The second approach is fully scalable and more efficient when m is close to n. Ultimately, the greatest efficiency is achieved with a rule prescribing which method to use in which situation, e.g. as a function of n and m that compares the number of combinations required for each method. Multisets and combinations are generated with a combinatorial mixed radix system algorithm (MIRSA) [30, 31].

$P_u(U, n)$ can also be defined recursively:

$$P_u(U, n) = \sum_{a \in U} \pi(a)\big[P_u(U, n-1) + P_u(U \backslash \{a\}, n-1)\big]. \tag{S.1}$$

This definition follows from Equation (2) and provides an essential simplification step that will allow us to derive a conditional probability of observed data given detection and recrudescence for a pair of samples and generalize it to any combination and number of undetected alleles and recrudescent strains.

S.2 Likelihood derivation

To translate observed unique alleles into full data and to detangle missingness and recrudescence, we need to combine combinatorics with tracking specific strains across loci (rows in $\mathbf{X}$, $\mathbf{Y}$) by fixing their indices. Starting with a single sample, let $\mathbf{D}$ be a random binary detection matrix such that

bioRxiv preprint doi: https://doi.org/10.1101/2025.04.28.651146; this version posted September 15, 2025. The copyright holder for this preprint (which was not certified by peer review) is the author/funder, who has granted bioRxiv a license to display the preprint in perpetuity. It is made available under aCC-BY 4.0 International license.

$P(D_{il} = 1) = 1 \; \forall i \neq k$ for a fixed index k of a single minor strain in the sample, $p_{kl} < 1 \; \forall l$. D_{il}, $i \neq k$, have a degenerate distribution and take a value of 1. k'th row of $\mathbf{D}$ can take values in $\{0, 1\}$. If $D_{kl} = 1$ for some locus l, alleles in U_l can be spread out across all n strains. If $D_{kl} = 0$, those alleles have to be in the other $n - 1$ strains, and an unobserved realization of X_{kl} could be any allele $a \in A_l$ with probability $\pi(a)$. Then

$$P(U_l \mid n, D_{kl} = 0) = \sum_{a \in A_l} \pi(a) P_u(U_l,\, n-1) = P_u(U_l,\, n-1) \underbrace{\sum_{a \in A_l} \pi(a)}_{1} = P_u(U_l,\, n-1)$$

In general, for any number of minor strains, $P(U_l \mid n, D_{\cdot l}) = P_u(U_l,\, \sum_{i=1}^{n} D_{il})$.

Next, we consider a pair of samples with a single recrudescent strain $i = 1$ (the strains in $\mathbf{X}$ and $\mathbf{Y}$ are ordered in such a way that recrudescent strains are in the top rows and their indices are matching in $\mathbf{X}$ and $\mathbf{Y}$), $IBD_{1l} = 1 \; \forall l$. For some locus l assume that all the alleles have been detected in both samples and that allele $a \in U_{xy}$, $U_{xy} = U_x \cap U_y$ is in a recrudescent strain. That means that remaining $n_x - 1$ strains in $\mathbf{X}$ contain either all of the alleles in $U_{x,l}$ or all of the alleles except allele a; the same goes for $n_y - 1$ strains in $\mathbf{Y}$. Therefore, when there is no missingness,

$$\begin{aligned} P(U_{x,l}, U_{y,l} \mid n_x, n_y, IBD_{1l} = 1) &= \sum_{a \in U_{xy}} \pi(a) \big[P_u(U_{x,l},\, n_x - 1) + P_u(U_{x,l} \backslash \{a\},\, n_x - 1)\big] \\ &\qquad \times \big[P_u(U_{y,l},\, n_y - 1) + P_u(U_{y,l} \backslash \{a\},\, n_y - 1)\big] \\ &= P_{xy}^{(1)}(U_{x,l}, U_{y,l},\, n_x, n_y), \end{aligned} \tag{S.2}$$

using $P_{xy}^{(1)}$ function defined in Equation (6). If $|U_{x,l}| = n_x$, an allele in a recrudescent strain cannot be in any other strains; in that case $P_u(U_{x,l},\, n_x - 1) = 0$. There is a similarity between Equations (S.1) (one sample) and (6) (pair of samples); Equation (6) however cannot be simplified further as the summation goes over the elements of U_{xy} only and not over all the elements of $U_{x,l}$ or $U_{y,l}$.

Adding missingness to this scenario, we first consider a single minor strain in each sample. At a locus, there could be no alleles missing in either sample, one sample with an undetected allele and another with detected, or both undetected. For minor strains j in the D0 sample and k in the DR sample (omitting conditioning on n_x, n_y in notation for brevity),

$$\begin{aligned} P(U_{x,l}, U_{y,l} \mid IBD_{1l} = 1) = \;& p_{x,jl} \; p_{y,kl} \; P(U_{x,l}, U_{y,l} \mid D_{x,jl} = 1, D_{y,kl} = 1, IBD_{1l} = 1) \\ +\;& p_{x,jl} \, (1 - p_{y,kl}) \, P(U_{x,l}, U_{y,l} \mid D_{x,jl} = 1, D_{y,kl} = 0, IBD_{1l} = 1) \\ +\;& (1 - p_{x,jl}) \; p_{y,kl} \; P(U_{x,l}, U_{y,l} \mid D_{x,jl} = 0, D_{y,kl} = 1, IBD_{1l} = 1) \\ +\;& (1 - p_{x,jl}) \, (1 - p_{y,kl}) \, P(U_{x,l}, U_{y,l} \mid D_{x,jl} = 0, D_{y,kl} = 0, IBD_{1l} = 1) \end{aligned}$$

To find $P(U_{x,l}, U_{y,l} \mid D_{x,jl}, D_{y,kl}, IBD_{1l})$, there are three main cases and six combinations altogether that need to be covered:

1. alleles detected in both samples;
2. allele detected in one sample, not detected in another:
    (a) an undetected allele is in a recrudescent strain;
    (b) an undetected allele is not in a recrudescent strain;

bioRxiv preprint doi: https://doi.org/10.1101/2025.04.28.651146; this version posted September 15, 2025. The copyright holder for this preprint (which was not certified by peer review) is the author/funder, who has granted bioRxiv a license to display the preprint in perpetuity. It is made available under aCC-BY 4.0 International license.

3. alleles undetected in both samples:
   (a) undetected alleles in both samples are in a recrudescent strain;
   (b) an undetected allele is in a recrudescent strain in one sample and not in a recrudescent strain in another;
   (c) undetected alleles in both samples are not in a recrudescent strain;

The first case with both alleles detected is equivalent to the one with no minor strains:

$$P(U_{x,l}, U_{y,l} \,|\, D_{x,1l} = 1, D_{y,1l} = 1, IBD_{1l} = 1) = P^{(1)}_{xy}(U_{x,l}, U_{y,l},\, n_x, n_y)$$

For the second case, suppose the allele in recrudescent strain $i = 1$ is missing in the DR sample. Using Equation (S.1),

$$\begin{aligned} P(U_{x,l}, U_{y,l} \,|\, D_{x,jl} = 1, D_{y,1l} = 0, IBD_{1l} = 1) &= \sum_{a \in U_{x,l}} \pi(a) \big[\, P_u(U_{x,l},\, n_x - 1) + P_u(U_{x,l} \backslash \{a\},\, n_x - 1)\big] \\ &\qquad \times P_u(U_{y,l},\, n_y - 1) \\ &= P_u(U_{x,l},\, n_x)\, P_u(U_{y,l},\, n_y - 1) \end{aligned} \tag{S.3}$$

When the minor strain with an undetected allele in the DR sample is not the recrudescent one ($k \neq 1$),

$$\begin{aligned} P(U_{x,l}, U_{y,l} \,|\, D_{x,jl} = 1, \underset{k>1}{D_{y,kl}} = 0, IBD_{1l} = 1) &= \sum_{a \in U_{xy}} \pi(a) \big[P_u(U_{x,l},\, n_x - 1) + P_u(U_{x,l} \backslash \{a\},\, n_x - 1)\big] \\ &\quad \times \Bigg[P_u(U_{y,l},\, n_y - 2) \underbrace{\sum_{b \in A_l} \pi(b)}_{1} + P_u(U_{y,l} \backslash \{a\},\, n_y - 2) \underbrace{\sum_{b \in A_l} \pi(b)}_{1}\Bigg] \\ &= P^{(1)}_{xy}(U_{x,l}, U_{y,l},\, n_x, n_y - 1) \end{aligned} \tag{S.4}$$

using Equation (6). Note that the index k is known and fixed across the loci, so when an allele Y_{kl} is undetected, that position needs to be excluded from combinatorial calculations in $P^{(1)}_{xy}$ function (hence $n_y - 2$ in the RHS of Equation (S.4)).

For the third case, when undetected alleles in both samples are in the recrudescent strain ($j = 1$, $k = 1$),

$$\begin{aligned} P(U_{x,l}, U_{y,l} \,|\, D_{x,1l} = 0, D_{y,1l} = 0, IBD_{1l} = 1) &= \sum_{a \in A_l} \pi(a) P_u(U_{x,l},\, n_x - 1)\, P_u(U_{y,l},\, n_y - 1) \\ &= P_u(U_{x,l},\, n_x - 1)\, P_u(U_{y,l},\, n_y - 1) \underbrace{\sum_{a \in A_l} \pi(a)}_{1} \\ &= P_u(U_{x,l},\, n_x - 1)\, P_u(U_{y,l},\, n_y - 1) \end{aligned} \tag{S.5}$$

When the minor strains with undetected alleles are the recrudescent strain in the D0 sample ($j = 1$) but not the recrudescent one in the DR sample ($k \neq 1$),

$$\begin{aligned} P(U_{x,l}, U_{y,l} \,|\, D_{x,1l} = 0, \underset{k>1}{D_{y,kl}} = 0, IBD_{1l} = 1) &= \sum_{a \in U_{y,l}} \pi(a) P_u(U_{x,l},\, n_x - 1) \\ &\quad \times \Bigg[P_u(U_{y,l},\, n_y - 2) \underbrace{\sum_{b \in A_l} \pi(b)}_{1} + P_u(U_{y,l} \backslash \{a\},\, n_y - 2) \underbrace{\sum_{b \in A_l} \pi(b)}_{1}\Bigg] \\ &= P_u(U_{x,l},\, n_x - 1)\, P_u(U_{y,l},\, n_y - 1) \end{aligned} \tag{S.6}$$

bioRxiv preprint doi: https://doi.org/10.1101/2025.04.28.651146; this version posted September 15, 2025. The copyright holder for this preprint (which was not certified by peer review) is the author/funder, who has granted bioRxiv a license to display the preprint in perpetuity. It is made available under aCC-BY 4.0 International license.

Finally, when undetected alleles are not in the recrudescent strain in both samples ($j \neq 1$, $k \neq 1$),

$$\begin{aligned}
P(U_{x,l}, U_{y,l} \,|\, \underset{j>1}{D_{x,jl}} = 0, \underset{k>1}{D_{y,kl}} = 0, IBD_{1l} = 1) &= \sum_{a \in U_{xy}} \pi(a) \Big[P_u(U_{x,l},\, n_x - 2) \underbrace{\sum_{b \in A_l} \pi(b)}_{1} + P_u(U_{x,l} \backslash \{a\},\, n_x - 2) \underbrace{\sum_{b \in A_l} \pi(b)}_{1} \Big] \\
&\times \Big[P_u(U_{y,l},\, n_y - 2) \underbrace{\sum_{b \in A_l} \pi(b)}_{1} + P_u(U_{y,l} \backslash \{a\},\, n_y - 2) \underbrace{\sum_{b \in A_l} \pi(b)}_{1} \Big] \\
&= P_{xy}^{(1)}(U_{x,l},\, U_{y,l},\, n_x - 1, n_y - 1)
\end{aligned} \tag{S.7}$$

So far, we have only considered a single minor strain in each sample and a single recrudescent strain (or, more precisely, a single pair of strains that are IBD at a locus since alleles can also be IBD as a result of a reinfection with a related strain). For a general case, i.e. a conditional probability $P(U_{x,l}, U_{y,l} \,|\, D_{x,\cdot l}, D_{y,\cdot l}, IBD_{\cdot l})$ where $D_{x,\cdot l}$, $D_{y,\cdot l}$, and $IBD_{\cdot l}$ are any binary sequences of lengths n_x, n_y, and $\min(n_x, n_y)$ respectively, note that the only strains that contribute to possible dependence between $U_{x,l}$ and $U_{y,l}$ are the ones where alleles are simultaneously IBD and detected in both samples; let $m \equiv \sum_{i1}^{\min(n_x,n_y)} D_{x,il}\, D_{y,il}\, IBD_{il}$ be the number of such strains. Undetected alleles, either IBD or not, factor into the conditional probability only through the second argument of $P_u(U,\, n)$ function, which makes intuitive sense (reducing the number of strains to which elements of U can be allocated) and is formally shown in Equations (S.3), (S.4), (S.5), (S.6), and (S.7). Therefore, the conditional probability only depends on m and the number of detected alleles in each sample, which can be summarized as follows:

$$P(U_{x,l}, U_{y,l} \,|\, D_{x,\cdot l}, D_{y,\cdot l}, IBD_{\cdot l}) = P_{xy}^{(m)} \left(U_{x,l}, U_{y,l}, \sum_{i=1}^{n_x} D_{x,il}, \sum_{j=1}^{n_y} D_{y,jl} \right)$$

To derive $P_{xy}^{(m)}(U_x, U_y,\, n_x, n_y)$, we divide strain indices in $\mathbf{X}$ and $\mathbf{Y}$ into three groups: matching indices for IBD and detected strains and remaining indices in each sample. In m strains of the first group, all alleles can be different, some can be the same, or all can be the same; they also do not need to include all the elements of U_{xy}, some of which could be matching by chance. The other two groups should contain all the remaining alleles for each sample not allocated to the first group and may or may not also contain alleles already present in the first group:

$$P_{xy}^{(m)}(U_{x,l}, U_{y,l},\, n_x, n_y) = \sum_{U' \subseteq U_{xy}} P_u(U',\, m) \sum_{U'' \subseteq U'} P_u(U_{x,l} \backslash U'',\, n_x - m) \sum_{U''' \subseteq U'} P_u(U_{y,l} \backslash U''',\, n_y - m)$$

Some $U' \subseteq U_{xy}$ in the first sum can produce terms that are equal to 0: if $|U'| > m$, $P_u(U',\, m) = 0$; an empty set, which is an element of a power set of U_{xy} will also result in a term of 0 if $m > 0$ since $P(\varnothing,\, m > 0) = 0$. Another constraint on the cardinality of $|U'|$ is imposed by the cardinality of its complements with respect to $U_{x,l}$ and $U_{y,l}$ as the number of alleles not included in U' should not exceed $n_x - m$ or $n_y - m$; if it does, all of the terms in the second or the third sum will be equal to 0. The cases with $U'' = \varnothing$ or $U''' = \varnothing$ however represent scenarios where all the IBD alleles are also present in the other strains and are not generally equal to 0. Using empty sets, we obtain a special case of $m = 0$:

$$\begin{aligned}
P_{xy}^{(0)}(U_{x,l}, U_{y,l},\, n_x, n_y) &= \underbrace{P_u(\varnothing,\, 0)}_{1}\, P_u(U_{x,l} \backslash \varnothing,\, n_x - 0)\, P_u(U_{y,l} \backslash \varnothing,\, n_y - 0) \\
&= P_u(U_{x,l},\, n_x)\, P_u(U_{y,l},\, n_y)
\end{aligned}$$

bioRxiv preprint doi: https://doi.org/10.1101/2025.04.28.651146; this version posted September 15, 2025. The copyright holder for this preprint (which was not certified by peer review) is the author/funder, who has granted bioRxiv a license to display the preprint in perpetuity. It is made available under aCC-BY 4.0 International license.

Another special case, $m = 1$, is covered in Equation (S.2) when there is no missingness; the same logic applies when alleles in some strains are undetected.

S.3 Background relatedness

Consider a base case where at most one strain $i = 1$ can be recrudescent: $P(R'_j = 1) = 0$ for $j = 2, \ldots, \min(n_x, n_y)$ and consequently $P(R = 1) = P(R'_1 = 1) = \theta$. Then

$$\begin{aligned}
P(\boldsymbol{U}_x, \boldsymbol{U}_y) = &\; P(R'_1 = 1)\, P(\boldsymbol{U}_x,\; \boldsymbol{U}_y \mid R'_1 = 1) \\
&+ P(R'_1 = 0)\, P(\boldsymbol{U}_x,\; \boldsymbol{U}_y \mid R'_1 = 0) \\
= &\; P(R'_1 = 1) \prod_{l=1}^{L} \Big[P(U_{x,l}, U_{y,l} \,|\, IBD_{1l} = 1)\, \underbrace{P(IBD_{1l} = 1 \,|\, R'_1 = 1)}_{1} \\
&\qquad + P(U_{x,l}, U_{y,l} \,|\, IBD_{1l} = 0)\, \underbrace{P(IBD_{1l} = 0 \,|\, R'_1 = 1)}_{0} \Big] \\
&+ P(R'_1 = 0) \prod_{l=1}^{L} \Big[P(U_{x,l}, U_{y,l} \,|\, IBD_{1l} = 1)\, \underbrace{P(IBD_{l} = 1 \,|\, R'_1 = 0)}_{r_{bg}} \\
&\qquad + P(U_{x,l}, U_{y,l} \,|\, IBD_{1l} = 0)\, \underbrace{P(IBD_{il} = 0 \,|\, R'_1 = 0)}_{1 - r_{bg}} \Big] \\
= &\; \theta \prod_{l=1}^{L} P(U_{x,l}, U_{y,l} \,|\, IBD_{1l} = 1) \\
&+ (1 - \theta) \prod_{l=1}^{L} \Big[P(U_{x,l}, U_{y,l} \,|\, IBD_{il} = 1)\, r_{bg} \\
&\qquad + P(U_{x,l}, U_{y,l} \,|\, IBD_{1l} = 0)\, (1 - r_{bg}) \Big],
\end{aligned}$$

using the fact that observed data depends on recrudescence through IBD only, i.e. $P(U_{x,l}, U_{y,l} \,|\, IBD_{il}, R'_i) = P(U_{x,l}, U_{y,l} \,|\, IBD_{il})$.

In Section 2.1.2 we defined r_{bg} on a strain level, which implies that every newly infecting strain can be related to an originally present strain with r_{bg} IBD on average. If multiple pairs of non-recrudescent strains are related between a pair of the D0 and DR samples, all the IBD combinations at a locus need to be accounted for. In addition, an assumption of independence of all interhost IBD variables at a locus would be required to avoid transitive dependencies. In practice, however, relatedness for a pair of samples is often estimated on an infection level. This interpretation can be easily accommodated in Aster by restricting interhost relatedness to a single pair of strains, which would allow infection-level estimates to be used for r_{bg} directly while decreasing the number of combinations required to calculate the likelihood.

S.4 Missing data

Missing data $U_{x,l} = \varnothing$ or $U_{y,l} = \varnothing$, i.e. no detected alleles at locus l in a sample, is a special case of realization of $D_{x,\cdot l}$ or $D_{y,\cdot l}$ that is actually observed (a sequence of 0's); another special observed case being a sequence of 1's, which would be inferred when $|U_{x,l}| = n_x$ (assuming no false positive

bioRxiv preprint doi: https://doi.org/10.1101/2025.04.28.651146; this version posted September 15, 2025. The copyright holder for this preprint (which was not certified by peer review) is the author/funder, who has granted bioRxiv a license to display the preprint in perpetuity. It is made available under aCC-BY 4.0 International license.

alleles). Intuitively, such locus provides no information as to recrudescence regardless of detected alleles in the other sample; we show this formally. Since $\sum_{i=1}^{n_{xy}} D_{x,il} D_{y,il} IBD_{il} = 0$ regardless of $\sum_{i=1}^{n_{xy}} IBD_{il}$, where $n_{xy} = \min(n_x, n_y)$, we use Equation (8) and $P(\varnothing,\, 0) = 1$ to get

$$\begin{aligned} P\big(U_{x,l} = \varnothing, U_{y,l} \,|\, n_x, n_y, IBD_{\cdot l}\big) &= P\Bigg(\sum_{j=1}^{n_x} D_{x,jl} = 0\Bigg) \sum_{D_{y,\cdot l} \in \mathcal{B}^{(n_y)}} P(D_{y,\cdot l})\, P_{xy}^{(0)}\Bigg(\varnothing, U_{y,l},\, 0, \sum_{i=1}^{n_y} D_{y,il}\Bigg) \\ &= \left[\prod_{j=1}^{n_x} (1 - p_{x,jl})\right] \sum_{D_{y,\cdot l} \in \mathcal{B}^{(n_y)}} P(D_{y,\cdot l})\, P_u(\varnothing,\, 0)\, P_u\Bigg(U_{y,l}, \sum_{i=1}^{n_y} D_{y,il}\Bigg) \\ &= \left[\prod_{j=1}^{n_x} (1 - p_{x,jl})\right] \sum_{D_{y,\cdot l} \in \mathcal{B}^{(n_y)}} P(D_{y,\cdot l})\, P(U_{y,l} \,|D_{y,\cdot l}) \\ &= \left[\prod_{j=1}^{n_x} (1 - p_{x,jl})\right] P(U_{y,l} \,|\, n_y), \end{aligned}$$

where $\mathcal{B}^{(n)}$ is a set of all the binary sequences of length n. Similarly,

$$P\big(U_{x,l}, U_{y,l} = \varnothing \,|\, n_x, n_y, IBD_{\cdot l}\big) = \left[\prod_{k=1}^{n_y} (1 - p_{y,kl})\right] P(U_{x,l} \,|\, n_x) \qquad \text{and}$$

$$P\big(U_{x,l} = \varnothing, U_{y,l} = \varnothing \,|\, n_x, n_y, IBD_{\cdot l}\big) = \left[\prod_{j=1}^{n_x} (1 - p_{x,jl})\right] \left[\prod_{k=1}^{n_y} (1 - p_{y,kl})\right],$$

the latter being a special case when no alleles are detected at locus l in either sample. The fact that $P\big(U_{x,l} = \varnothing, U_{y,l} \,|\, n_x, n_y, \sum_{i=1}^{n_{xy}} IBD_{il} > 0\big) = P\big(U_{x,l} = \varnothing, U_{y,l} \,|\, n_x, n_y, \sum_{i=1}^{n_{xy}} IBD_{il} = 0\big)$ means that recrudescence and no recrudescence are given the same weight, which can be factored out of the likelihood (of the form $\theta\, C_1 + (1 - \theta)\, C_0$) and thus does not affect the shape of the log-likelihood function. This confirms that loci with no data in at least one of the samples can be ignored without introducing any bias.

Using similar logic, consider another special case that warrants attention and might be potentially problematic - completely undetected strains if they are recrudescent. Let strain $i = 1$ be possibly recrudescent and let $p_{x,1l} = 0 \;\forall\, l$; assume no other strains can be recrudescent or IBD. Then $D_{x,1l} = 0$, $\sum_{i=1}^{n_{xy}} D_{x,il} D_{y,il} IBD_{il} = 0 \;\forall\, l$, and

$$P(U_{x,l}, U_{y,l} \,|\, n_x, n_y, IBD_{1l}) = P(U_{x,l} \,|\, n_x)\, P(U_{y,l} \,|\, n_y)$$

regardless of the value of IBD_{1l}. Since that is the case for all the loci, $P(\boldsymbol{U}_x, \boldsymbol{U}_y \,|\, R = 1) = P(\boldsymbol{U}_x, \boldsymbol{U}_y \,|\, R = 0)$, which implies that recurrence classification cannot be performed with any degree of certainty, and the individual essentially does not contribute to failure rate estimation (apart from censoring). This situation presents a potential source of bias: incorrectly assuming no recrudescent strains to be completely missing might lead to underestimation of drug failure, and always assuming that there is an undetected recrudescent strain will never allow a recurrence to be classified as a new infection. Fortunately, this is not likely to occur commonly; if available, an estimate of the probability of this occuring could be incorporated into the framework.

bioRxiv preprint doi: https://doi.org/10.1101/2025.04.28.651146; this version posted September 15, 2025. The copyright holder for this preprint (which was not certified by peer review) is the author/funder, who has granted bioRxiv a license to display the preprint in perpetuity. It is made available under aCC-BY 4.0 International license.

S.5 Supplementary Figures

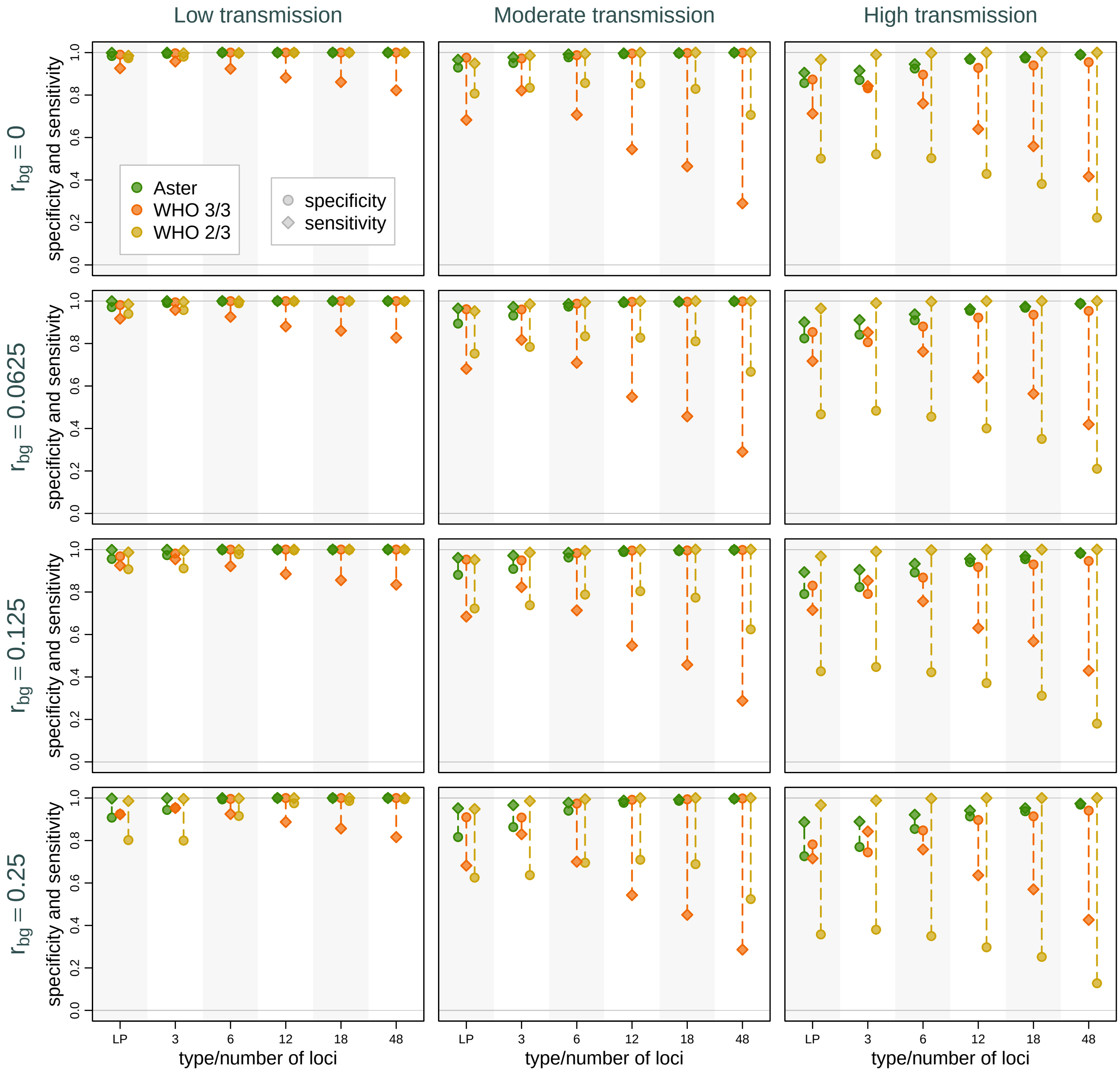

Figure S.1: Sensitivity and specificity of Aster and match-counting algorithms across trasmission intensity levels, genotyping panels, and background relatedness (r_{bg}) levels. Dotted lines mark the difference between sensitivity and specificity for a specific method in a setting; longer lines imply greater imbalance and potentially greater bias in failure rate estimation. Detection probability was set to 0.9.

bioRxiv preprint doi: https://doi.org/10.1101/2025.04.28.651146; this version posted September 15, 2025. The copyright holder for this preprint (which was not certified by peer review) is the author/funder, who has granted bioRxiv a license to display the preprint in perpetuity. It is made available under aCC-BY 4.0 International license.

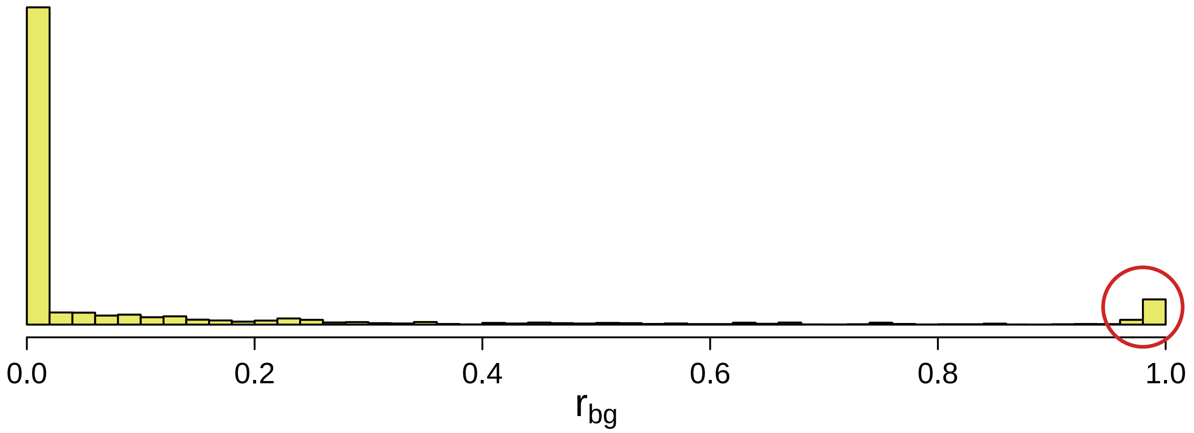

Figure S.2: Histogram of pairwise sample relatedness in Asayita, Ethiopia, estimated using Dcifer [30]. A red circle highlights an unusually high proportion of sample pairs that were highly related.

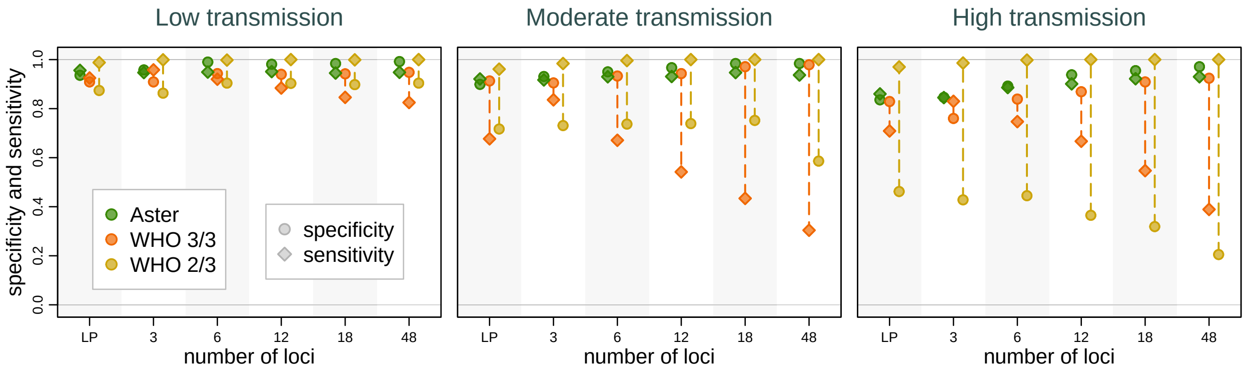

(a) True value of r_{bg} (usually unknown in practice) used as input to Aster for each pair of samples

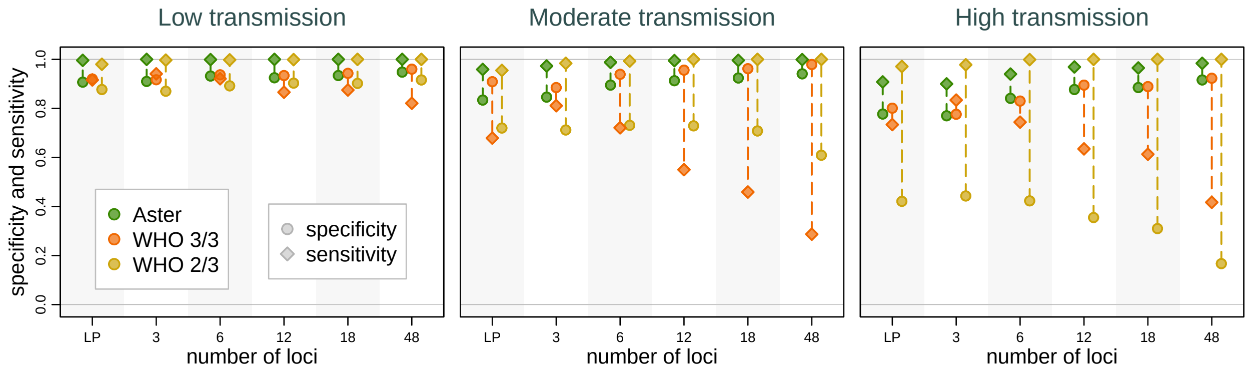

(b) A distribution of r_{bg} used as input to Aster for each pair of samples

Figure S.3: Classification performance of Aster and match-counting algorithms across genotyping panels and transmission intensities from simulations using an empirical distribution of r_{bg} from Asayita, Ethiopia. Detection probability was set to 0.9.

bioRxiv preprint doi: https://doi.org/10.1101/2025.04.28.651146; this version posted September 15, 2025. The copyright holder for this preprint (which was not certified by peer review) is the author/funder, who has granted bioRxiv a license to display the preprint in perpetuity. It is made available under aCC-BY 4.0 International license.

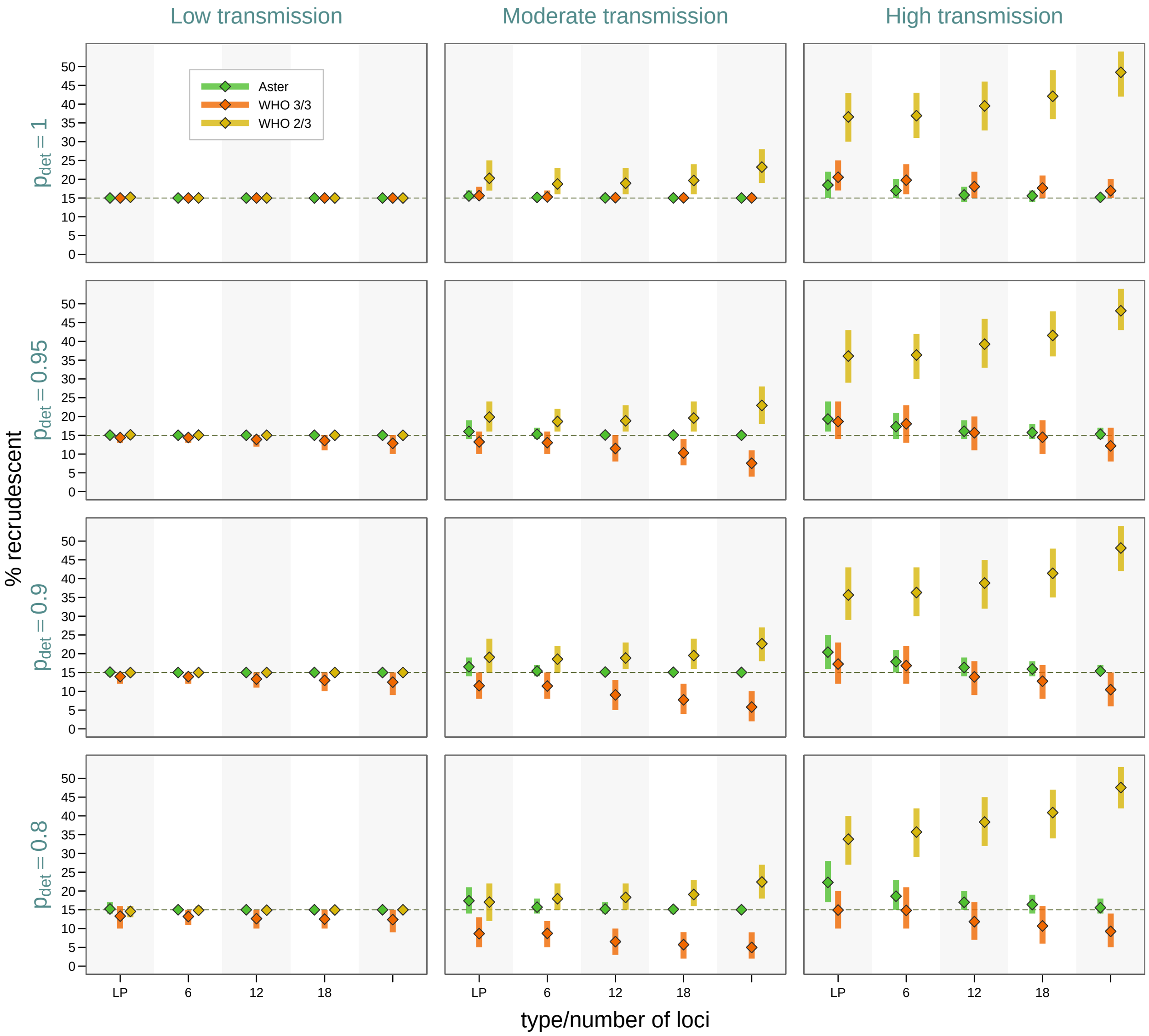

Figure S.4: Study-level estimates across transmission intensity levels, genotyping panels, and detection probabilities (p_{det}). The vertical bars represent a 95% range (0.025 to 0.975 quantiles) of the results, the diamond symbols represents a mean. Proportion of recrudescent events was fixed at 0.15 and background relatedness was 0.

bioRxiv preprint doi: https://doi.org/10.1101/2025.04.28.651146; this version posted September 15, 2025. The copyright holder for this preprint (which was not certified by peer review) is the author/funder, who has granted bioRxiv a license to display the preprint in perpetuity. It is made available under aCC-BY 4.0 International license.

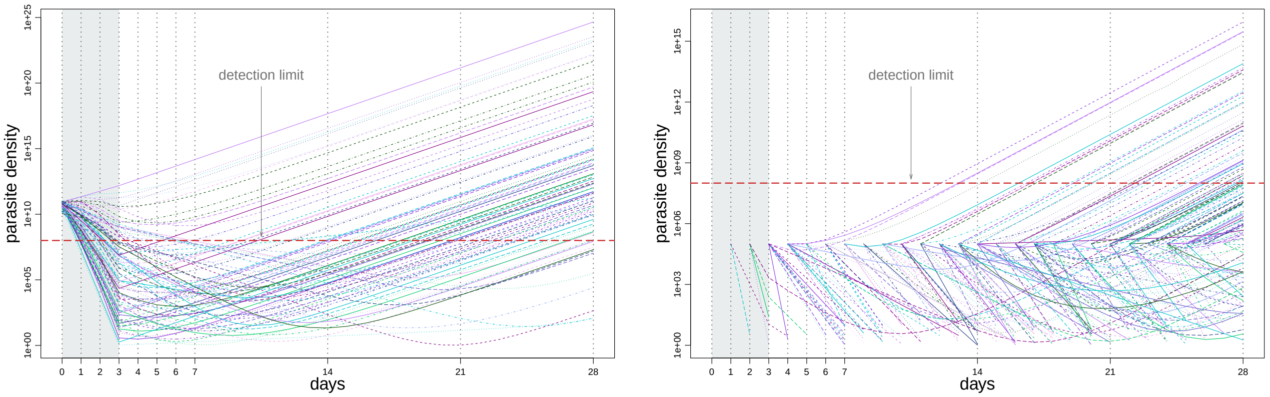

(a) Recrudescent strains **(b)** Newly acquired strains (introduced at 10^5 density)

Figure S.5: Simulated per-strain parasite density dynamics from PK/PD model. Vertical dotted lines represent time points at which the sample parasite density is measured; shaded area represents a treatment period. A dotted red horizontal line indicates a per-sample detection limit.

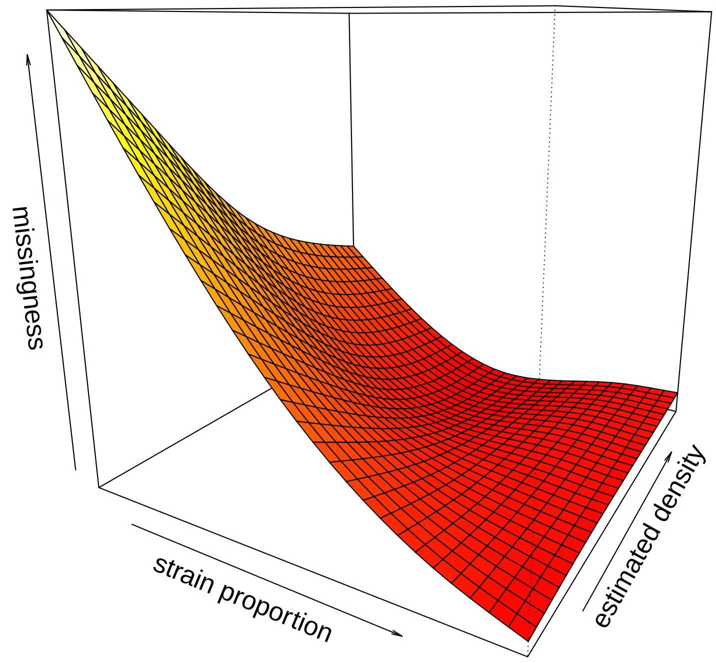

Figure S.6: A model fit to predict detection probability from sample parasite density and within-sample strain proportion using empirical data from mixed-strain controls taken from [24]. A general additive model (GAM) with full tensor product smooth was used for the fit, with parasite density and proportion on the log scale. Smaller within-host proportion and overall parasite density resulted in more undetected alleles.

bioRxiv preprint doi: https://doi.org/10.1101/2025.04.28.651146; this version posted September 15, 2025. The copyright holder for this preprint (which was not certified by peer review) is the author/funder, who has granted bioRxiv a license to display the preprint in perpetuity. It is made available under aCC-BY 4.0 International license.

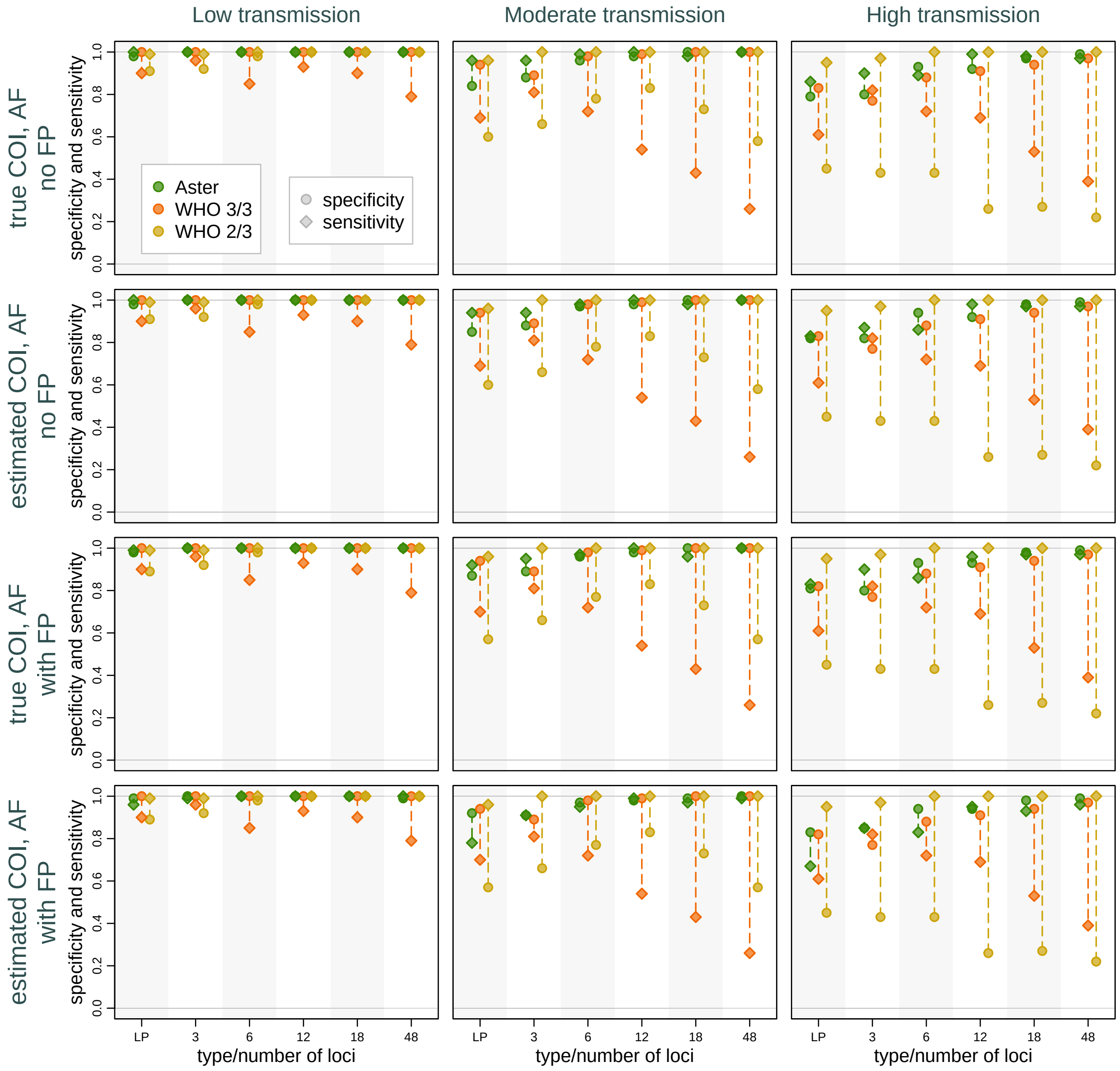

Figure S.7: Sensitivity and specificity of Aster and match-counting algorithms exploring the effect of using estimated vs true COI and population allele frequencies (AF) and of adding simulated false positive alleles (FP). Allele frequencies were estimated from 100 D0 samples. False positive rates were 0.1 for the length polymorphism (LP) panel and 0.02 for the amplicon sequencing panels.

bioRxiv preprint doi: https://doi.org/10.1101/2025.04.28.651146; this version posted September 15, 2025. The copyright holder for this preprint (which was not certified by peer review) is the author/funder, who has granted bioRxiv a license to display the preprint in perpetuity. It is made available under aCC-BY 4.0 International license.

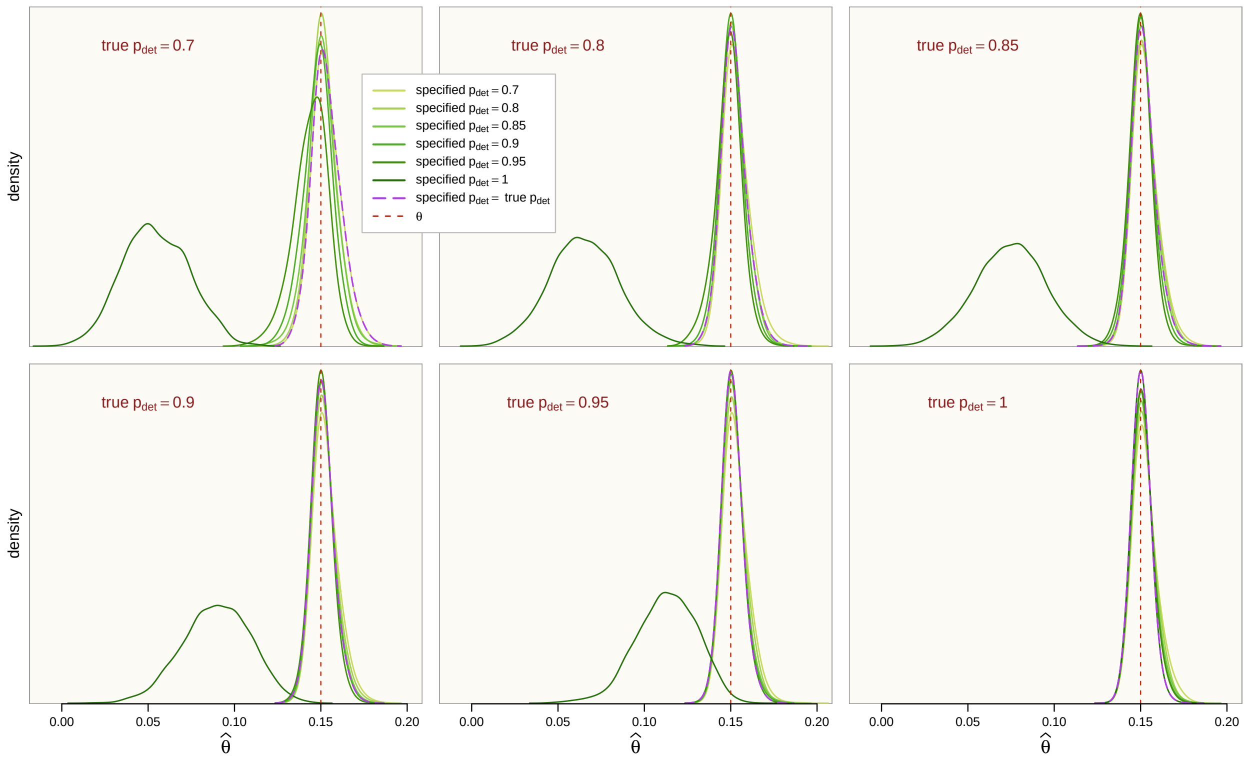

Figure S.8: Distribution of failure rate estimates with Aster when using misspecified allele detection probability (p_{det}). Each panel features one value of true detection probability evaluated with a range of specified values, one of which matched the true probability (purple dotted line marking the corresponding density). Study-level simulation settings were moderate transmission intensity, failure rate (θ) of 0.15 (vertical red line), and 0 background relatedness. A 12-locus MAD^{4}HatTeR panel was used for the simulations.
